# Aberrant promoter methylation contributes to *LRIG1* silencing in basal/triple-negative breast cancer

**DOI:** 10.1038/s41416-022-01812-8

**Published:** 2022-04-19

**Authors:** Maxine Umeh-Garcia, Henriette O’Geen, Catalina Simion, Melanie Hayden Gephart, David J. Segal, Colleen A. Sweeney

**Affiliations:** 1 Department of Biochemistry and Molecular Medicine, University of California, Davis, CA USA; 2 Department Neurosurgery, Stanford University, Stanford, CA USA; 3 Genome Center, University of California, Davis, CA USA

**Keywords:** Breast cancer, DNA methylation, Gene silencing, Tumour-suppressor proteins

## Abstract

**Background:**

LRIG1, the founding member of the LRIG (leucine-rich repeat and immunoglobulin-like domain) family of transmembrane proteins, is a negative regulator of receptor tyrosine kinases and a tumour suppressor. Decreased *LRIG1* expression is consistently observed in cancer, across diverse tumour types, and is linked to poor patient prognosis. However, mechanisms by which *LRIG1* is repressed are not fully understood. Silencing of *LRIG1* through promoter CpG island methylation has been reported in colorectal and cervical cancer but studies in breast cancer remain limited.

**Methods:**

In silico analysis of human breast cancer patient data were used to demonstrate a correlation between DNA methylation and *LRIG1* silencing in basal/triple-negative breast cancer, and its impact on patient survival. *LRIG1* gene expression, protein abundance, and methylation enrichment were examined by quantitative reverse-transcription PCR, immunoblotting, and methylation immunoprecipitation, respectively, in breast cancer cell lines in vitro. We examined the impact of global demethylation on *LRIG1* expression and methylation enrichment using 5-aza-2’-deoxycytidine. We also examined the effects of targeted demethylation of the *LRIG1* CpG island, and transcriptional activation of *LRIG1* expression, using the RNA guided deadCas9 transactivation system.

**Results:**

Across breast cancer subtypes, *LRIG1* expression is lowest in the basal/triple-negative subtype so we investigated whether differential methylation may contribute to this. Indeed, we find that *LRIG1* CpG island methylation is most prominent in basal/triple-negative cell lines and patient samples. Use of the global demethylating agent 5-aza-2’-deoxycytidine decreases methylation leading to increased *LRIG1* transcript expression in basal/triple-negative cell lines, while having no effect on *LRIG1* expression in luminal/ER-positive cell lines. Using a CRISPR/deadCas9 (dCas9)-based targeting approach, we demonstrate that TET1-mediated demethylation (Tet1-dCas9) along with VP64-mediated transcriptional activation (VP64-dCas9) at the CpG island, increased endogenous *LRIG1* expression in basal/triple-negative breast cancer cells, without transcriptional upregulation at predicted off-target sites. Activation of *LRIG1* by the dCas9 transactivation system significantly increased LRIG1 protein abundance, reduced site-specific methylation, and reduced cancer cell viability. Our findings suggest that CRISPR-mediated targeted activation may be a feasible way to restore *LRIG1* expression in cancer.

**Conclusions:**

Our study contributes novel insight into mechanisms which repress *LRIG1* in triple-negative breast cancer and demonstrates for the first time that targeted de-repression of *LRIG1* in cancer cells is possible. Understanding the epigenetic mechanisms associated with repression of tumour suppressor genes holds potential for the advancement of therapeutic approaches.

## Background

LRIG1 is a member of the “LRIG” family of single-pass transmembrane proteins, which also includes LRIG2 and LRIG3 [1, 2]. LRIG1 was identified as a negative regulator of various cell surface receptors, including ERBB [3, 4], PDGFR-A [5], MET [6], RET [7, 8] and TRKB [9] receptor tyrosine kinases. In 2012, LRIG1 was classified as a tumour suppressor, with genetic ablation leading to increased expression of ErbB receptors and the development of highly penetrant duodenal adenomas [10]. More recently, LRIG1 was reported to function as a haploinsufficient tumour suppressor in a PDGF-induced experimental glioma model, in part through negative regulation of MET [11].

Several studies have demonstrated decreased expression of *LRIG1* in cancer, including lung [12, 13], breast [14, 15] and head and neck cancer [16], among others [17]. Across diverse cancer types, high expression of *LRIG1* is associated with superior patient survival, in agreement with its tumour suppressor function [2, 17, 18]. This includes breast cancer, where low *LRIG1* expression has been linked to decreased relapse-free, and distant metastasis-free, survival [14, 15]. As an ERα target gene, *LRIG1* is most highly expressed in ER-positive luminal disease relative to other breast cancer subtypes [14], with the lowest expression observed in basal/triple-negative breast cancers (TNBC) [19, 20]. The mechanisms which contribute to *LRIG1* downregulation in breast cancer are not fully understood but are known to include ERBB2-mediated repression [14] and loss of gene copy number, which conveys persistent risk of relapse in patients typically considered low-risk [15]. In the context of cancer, DNA hypermethylation at the promoter of tumour suppressor genes is a key mechanism of downregulation and is recognised as a cancer hallmark in many tumour types [21]. Interestingly, hypermethylation of the *LRIG1* promoter region has been reported in colorectal cancer [22] as well as cervical cancer, where it was found to correlate with decreased progression-free survival [23]. However, studies on *LRIG1* gene methylation in breast cancer remain limited.

Since persistence of *LRIG1* expression is associated with superior patient survival across cancer types and because prior studies have shown that *LRIG1* is epigenetically and transcriptionally regulated, we reasoned that transcriptional reactivation of *LRIG1* expression in cancer cells was feasible. The clustered regularly interspaced short palindromic repeats (CRISPR) and CRISPR-associated protein 9 (Cas9) system has provided an efficient method for targeted gene activation. In the CRISPR/Cas9 system, endonuclease Cas9 is targeted to a specific DNA sequence by a single guide RNA (sgRNA) and subsequently induces double-stranded DNA cleavage [24]. A mutation of the catalytic domains of Cas9, yielding a deadCas9 (dCas9), maintains Cas9’s DNA-honing ability without inducing DNA breaks; and provides an ideal DNA targeting protein complex [25]. Recently, dCas9 fusions with the catalytic domains of a variety of effector proteins, such as ten-eleven translocation dioxygenase 1 (TET1), four tandem copies of Herpes Simplex Viral Protein 16 (VP64), and Krüppel-associated box (KRAB) have gained significance as candidate complexes that can demethylate, activate, or inactivate genes of interest, respectively [24].

We demonstrate that *LRIG1* methylation is significantly increased in breast tumours compared to normal tissue, with a strong inverse correlation between *LRIG1* mRNA expression and CpG island methylation. Interestingly, we find that methylation is most robust in the basal/TNBC subtype of breast cancer, both in patient samples and in cell lines representative of this subtype. Use of the global demethylating agent, 5-aza-2’-deoxycytidine (ADC), in TNBC cell lines leads to a significant increase in *LRIG1* mRNA expression aligning with a significant decrease in methylation. To directly implicate DNA methylation in *LRIG1* silencing, we take a targeted approach in which the catalytic domain of DNA hydroxymethyltransferase TET1 is fused to dCas9, and targeted to the *LRIG1* CpG island. We show, for the first time, that targeted demethylation causes significant reactivation of *LRIG1* gene expression and LRIG1 protein abundance, as well as reduces DNA methylation at the CpG island. We also demonstrate that co-expression of Tet1 and transcriptional activator, VP64, using the CRISPR/dCas9 system significantly amplifies many of these processes.

## Methods

### Cell culture

BT549 (Cat# HBT-122), HCC1937 (Cat# CRL-2336), MDA-MB-231 (Cat# HTB-26), MCF7 (Cat# HTB-22), T47D (Cat# HTB-133), ZR75-1 (Cat# CRL-1500) and HCT116 (Cat# CCL-247) cells were obtained from American Type Culture Collection (ATCC, Manassas, VA, USA) and maintained as recommended. Cell lines were authenticated by short tandem repeat profiling through the University of Arizona Genetics Core and tested for mycoplasma contamination using the MycoAlert PLUS Mycoplasma Detection Kit (Cat# LT37-701, Lonza, Hayward, CA, USA). All cells tested negative for mycoplasma contamination. Cells were used for 6–8 passages, after which they were replaced with a cryopreserved stock.

### ADC and panobinostat treatment

Cells were seeded in a 12-well plate at 7.5 × 10^5^ cells per well 24 h prior to treatments. Cells were treated with 5-aza-2’-deoxycytidine (ADC) (Cat# A3656, Sigma-Aldrich, St. Louis, MO, USA) at indicated concentrations for 96 h, with culture media being replaced every 24 h. Cells were treated with 100 μM Panobinostat (Cat# S1030, SelleckChem, Houston, TX, USA) for 24 h. For the combination experiment, cells were treated with 10 μM ADC for 96 h, with 100 μM Panobinostat added in the last 24 h. On completion of treatments, media was aspirated from culture wells, cells were rinsed with 1× PBS, and frozen at −80 °C until RNA isolation was performed.

### RNA extraction and reverse-transcription quantitative PCR (RT-qPCR)

RNA was collected using the PureLink RNA Mini Kit (Cat# 121183018, Thermo Fischer Scientific, Waltham, MA, USA) or RNeasy Mini Kit (Cat# 74104, QIAGEN, Germantown, MD, USA) according to the manufacturer’s protocols. High-Capacity cDNA Reverse Transcription Kit (Cat# 4368814, Thermo Fischer Scientific) or Iscript Reverse Transcription Supermix (Cat# 1708841, Bio-Rad, Hercules, CA, USA) was used to convert 0.5–1 μg of total RNA to cDNA. Quantitative PCR amplifications were conducted in a CFX96 real-time PCR system (Bio-Rad) using TaqMan probes (Life Technologies, Carlsbad, CA, USA) for *LRIG1* (Hs00394267_m1), *BASP1* (Hs00932356_s1), *CTNND2* (Hs00181643_m1), *FBXO15* (Hs00380856_m1), *NPHS1* (Hs00190446_m1), *KCNIP1* (Hs01557317_m1), *GUSB* (Hs99999908_m1), *ACTB* (Hs99999903_m1), *GAPDH* (Hs99999905_m1) and Taqman Fast Advanced Master Mix (Cat# 4444557, Thermo Fischer Scientific) or Universal SYBR Green Supermix (Cat# 1725120, Bio-Rad). Analysis was performed by the comparative Ct method under the following cycling conditions: 3 min at 95 °C, 40 cycles of 10 s at 95 °C and 30 s at 55 °C. Relative abundance was determined from the Ct values using the 2^-ΔΔCt^ method after normalisation to *GAPDH* (additional reference genes, *ACTB* and *GUSB*, were examined to ensure changes in *LRIG1* transcript levels were biologically accurate and not due to changes in *GAPDH* transcript levels, Supplemental Fig. 9).

### In silico analysis of DNA methylation

The UCSC Genome Browser was used to perform a qualitative in silico analysis of methylation levels across the *LRIG1* CpG island using the DNA Methylation by Reduced Representation Bisulfite Seq (RRBS) track. This track was produced as part of the ENCODE project by the Lab of Dr. Richard Myers at the HudsonAlpha Institute for Biotechnology [26] and reports the percentage of DNA molecules that exhibit cytosine methylation at more than 500,000 specific CpG dinucleotide sites in the human genome using bisulfite sequencing. For each assayed CpG, the percentage of methylated sequencing reads is reported. Methylation is represented as an 11-colour gradient with red = 100%, yellow = 50%, and green = 0% of sequenced reads methylated; respectively.

### Methylated DNA immunoprecipitation (MeDIP)

Cells were seeded in 10-cm plates and grown to confluency. Genomic DNA was extracted by incubation of cells with digestion buffer (100 mM NaCl, 10 mM Tris-Cl, pH 8.0, 25 mM EDTA, pH 8.0, 0.5% SDS) and 0.1 mg/mL Proteinase K (Cat# AM2548, Thermo Fischer Scientific) for 12–18 hr at 50 °C. DNA was purified using phenol–chloroform, precipitated and dissolved in TE to a final concentration of 200 ng/μL. Genomic DNA was sonicated to an average size of 400 bp using the BioRuptor NGS (Diagenode, Denville, NJ, USA) and denatured for 10 min at 95 °C and immediately transferred on ice. In total, 2 μg of fragmented DNA and 2 μg of 5-methylcytosine antibody (Cat# 39649, Active Motif, Carlsbad, CA, USA) were added to 500 μL IP dilution buffer (16.7 mM Tris-Cl pH 8.1, 1.2 mM EDTA pH 8, 167 mM NaCl, 0.01% SDS, 1.1% Triton X-100) and immunoprecipitation of methylated DNA was performed by gentle rotation for overnight at 4 °C. After incubation for 1 hr with 2 μg Rabbit Anti-Mouse IgG (Cat# 55436, MP Biomedical, Irvine, CA, USA), immunoprecipitates were washed two times with IP Wash Buffer I (50 mM Tris-Cl pH 8.0, 150 mM NaCl, 1% NP40, 0.25% deoxycholic acid, 2 mM EDTA pH 8.0), followed by three washes with IP Wash Buffer II (100 mM Tris-Cl pH 8.0, 500 mM LiCl, 1% NP40, 1% deoxycholic acid), and one wash with IP Wash Buffer III (100 mM Tris-Cl pH 8.0, 500 mM LiCl, 1% NP40, 1% deoxycholic acid, 150 mM NaCl). DNA was eluted for 30 min with elution buffer (50 mM NaHCO_3_, 1% SDS, 0.1 mg/mL Proteinase K), purified using phenol/chloroform, and precipitated using ethanol according to MeDIP Protocol (Active Motif). The resulting DNA pellet was dissolved in 50 μL TE. Standard PCR reactions were performed using 1 μL of the immunoprecipitated DNA or 5% input control DNA using GoTaq polymerase (Cat# M3001, Promega, Madison, WI, USA). PCR products were separated by electrophoresis through 2% agarose gels and visualised using ethidium bromide. For qPCR analysis, 1.5 μL of immunoprecipitated DNA or 5% input control DNA was amplified with SYBR FAST mastermix (Cat# KK4600, KAPA Biosystems, Wilmington, MA, USA) or Universal SYBR Green Supermix (Cat# 1725120, Bio-Rad) using the CFX96 real-time PCR system (Bio-Rad) according to the manufacturer’s recommendations. MeDIP enrichment was calculated relative to input samples using dCq = Cq[MeDIP]-Cq[input]. MeDIP primer: LRIG1 promoter: forward 5’-GGACTGTGAGGACCCGAAC-3’, reverse 5’-GCCGCAGAGAGAACTTGG-3’, LRIG1 5’UTR: forward 5’-AAAGGGCGGCACTCACAG-3’, reverse 5’-CTGGGGACTCGCTGGACT-3’, SNRPN: forward 5’-GCAAAACAGCCAGAACGTGAA-3’, reverse 5’-GCACACGAGCAATGCCAGTAT-3’.

### Western blotting

Total protein was extracted from cells using RIPA lysis buffer [50 mM Tris (pH 7.4), 150 mM NaCl, 0.1% SDS, 1% Triton X-100, 0.5% sodium deoxycholate]. After incubation on ice, cells were scraped from wells, lysates were vortexed and centrifuged at 13,000 × *g* for 15 min. Lysate supernatants were placed into new microcentrifuge tubes and protein concentrations were determined using Pierce BCA Protein Assay Kit (Cat# 23225, Thermo Fischer Scientific). A unit of 10–15 µg of cell lysate was denatured in 6× Laemmli sample buffer [50 mM Tris-HCl (pH 6.8), 2% SDS, 10% glycerol, 0.25% b-mercaptoethanol, and bromophenol blue (1 mg/mL)] at 100 °C for 5 min. Two times Laemmli sample buffer was added to samples to bring to a final volume of ~30–40 µL. Samples were separated on an 8% SDS-polyacrylamide gel or 4–12% Novex Tris-Glycine gel (Cat# XP04120BOX, Thermo Fischer Scientific) and then transferred to the nitrocellulose membrane (Cat# 1620115, MilliporeSigma, Burlington, MA, USA). Membranes were blocked with 5% non-fat dry milk in TBST (Tris-buffered saline containing 0.05% Tween 20) and incubated with either anti-LRIG1 (Cat# 12752, Cell Signaling Technologies, Danvers, MA, USA), anti-tubulin (Cat# T5168, Sigma-Aldrich), or anti-β-Actin (AC-15, Cat# A1978, Sigma-Aldrich) primary antibodies overnight at 4 °C. After incubation, membranes were washed with TBST and then incubated with horseradish peroxidase-conjugated secondary antibodies for 1 hr at room temperature. Chemiluminescence signals were visualised using Pierce ECL (Cat# 32106, Thermo Fischer Scientific) on an Alpha Innoteceh Digital Imaging Station. Blot images are a representation of three technical replicates from at least two independent experiments. Western blot densitometry for dCas9 assay was determined using Image J. Inverted protein band value or inverted membrane background value was expressed as 255 – X, where X is the mean grey value recorded by Image J. Net band values were determined by deducting the inverted membrane background values from the inverted protein band values. Relative protein quantification was determined by taking the net band value of LRIG1 over the net band value of Actin loading control for each lane.

### CRISPR-deadCas9 plasmids

The catalytic domain of mouse Tet1 (NP_001240786.1; aa 1367–2039), obtained by PCR amplification from plasmid ZF_B_-TET1CD. ZF_B_-TET1CD, was a kind gift from Marianne Rots [27]. Overhangs compatible with Gibson cloning were introduced during PCR amplification and TET1CD was then cloned into the KpnI digested dCas9 cloning vector (Addgene plasmid #100091) [28]. The resulting Tet1-dCas9 plasmid is available from Addgene (plasmid #136650). Similarly, plasmid VP64-dCas9 (Addgene plasmid #177171) was created by Gibson cloning of the VP64 activation domain into KpnI digested dCas9 cloning vector. pcDNA-dCas9-p300 Core was a gift from Charles Gersbach (Addgene plasmid #61357) [29].

### sgRNA target design and off-target identification

Guide RNA (sgRNA) target sequences were designed using CHOPCHOP [30]. Only the highest scoring target sequences were chosen. Selected sgRNAs were cloned as G-N19 into AflII-digested gRNA cloning vector using Gibson assembly (Addgene plasmid #41824) [31]. sgRNA sequences are listed in Supplemental Table S2. Off-target analysis of CRISPR sgRNAs was performed using the CCTop off-target prediction tool (https://cctop.cos.uni-heidelberg.de:8043) [32]. Briefly, 20 bp spacer sequences for sgRNAs candidates 5, 6, 12, 13 and 14 (without PAM sequences) were used as the query with hg19 as the reference genome for canonical SpCas9 PAM sites (NGG). The algorithm was executed using five or less total mismatches, a maximum core length of 12, and two or less core mismatches. The list of off-target genes for sgRNA 12, 13 and 14 was overlapped to identify collective off-targets of sgRNA Combination 1 (sgC1). In a similar manner, off-targets for sgRNA 5 and 6 were overlapped to identify collective sgRNA Combination 2 (sgC2) off-targets.

### Transient transfection of CRISPR/deadCas9

For CRISPR-dCas9-based transactivation of *LRIG1* by RT-qPCR and targeted DNA methylation analysis, 2 × 10^5^ cells were seeded in each well of a 12-well plate 18–24 h prior to transfection. Cells were transfected with a total mixture of 1.2 μg plasmid DNA per well (0.3 μg equimolar pooled sgRNAs, 0.7 μg equimolar pooled dCas9 complexes and 0.2 μg pBABE-puro (Addgene #1764) for Puromycin selection) using Lipofectamine 3000 and P3000 according to the manufacturer’s protocol. For western blotting, 5 × 10^5^ cells were seeded in each well of six-well plates and a total of 3.0 μg of plasmid DNA per well was used for transfection (0.75 μg equimolar pooled sgRNAs, 1.75 μg equimolar pooled dCas9 complexes and 0.5 μg pBABE-puro). For MTS cell viability, 1 × 10^4^ cells were seeded in each well of 96-well plates and a total of 0.12 μg plasmid DNA per well was used for transfection (0.03 μg equimolar pooled sgRNAs, 0.07 μg equimolar pooled dCas9 complexes and 0.02 μg pBABE-puro).

Transfection complexes were removed after 6–8 h and replaced with complete culture medium. 24 h post-transfection, culture medium was replaced with media containing 1–2 μg/mL Puromycin for 48 h to select for transfected cells. Untreated cells were seeded alongside transfected cells for each experiment and treated with Puromycin as a control for antibiotic selection. Following Puromycin selection, wells were gently rinsed twice with ice-cold 1× PBS to remove dead/non-transfected cell populations, and RNA extracted for RT-qPCR, lysed in RIPA for western blotting, or subjected to an MTS assay for cell viability.

### Cell viability

Viability was evaluated using the CellTiter 96^®^ AQueous One Solution Reagent (Cat# G3582, Promega), containing a tetrazolium compound [3-(4,5-dimethylthiazol-2-yl)-5-(3-carboxymethoxyphenyl)-2-(4-sulfophenyl)-2H-tetrazolium, inner salt; MTS] and an electron coupling reagent (phenazine ethosulfate; PES), per the manufacturer’s instructions.

### Targeted DNA methylation analysis

Genomic DNA was extracted from three independent experiments using the Quick-DNA Miniprep Kit (Cat# D3024, Zymo Research, Irvine, CA, USA) and 250–500 ng genomic DNA was bisulfite converted using the EZ DNA Methylation-Lightning Kit (Cat# D5030, Zymo Research). Bisulfite-Sequencing PCR (BSP) amplification of 25–50 ng of bisulfite converted ssDNA was carried out with ZymoTaq DNA polymerase (Cat# E2002, Zymo Research) according to the manufacturer’s instructions. MethPrimer 2.0 (http://www.urogene.org/methprimer2/) was used to design BSP primers with the degenerate primer parameter (LRIG1-BSP-F 5’-YGAGTTTTTAGYGTAAGTGTAGG-3’, LRIG1-BSP-R 5’-GTTRGAATCCTCACAATCCC-3’). Unique 6-nucleotide barcodes were added to the 5’-end of the forward primer sequence. Amplicons were then purified using the QIAquick PCR purification Kit (Cat# 28104, QIAGEN) and equal amounts were pooled. Library preparation and PE150 sequencing (CRISPR sequencing) were performed by the CCIB DNA Core Facility at Massachusetts General Hospital (Cambridge, MA). Sequence read files were demultiplexed and forward and reverse reads were merged into a single long read using FLASH2 [33]. Processed FASTQ files were aligned, and cytosine methylation states determined using Bismark [34]. All samples used for downstream analysis had a mapping efficiency of >99%. Percent methylation across 30 CG dinucleotides (CpG) sites spanning the BSP region (see Fig. 5), was determined by [% = (M/(M + U)) * 100], where M denotes methylated signal intensity and U unmethylated signal intensity, respectively. For each cell line, normalised percent methylation was determined after background subtraction of no treatment control percent methylation values.

### In silico analysis of human breast cancer data

The results of computational analysis are based on data generated by The Cancer Genome Atlas (TCGA) Research Network or NCBI Gene Expression Omnibus (GEO). Beta values (β-values) are the estimate of methylation level using the ratio of methylated and unmethylated intensities. β-values range from 0 to 1, with 0 being fully unmethylated and 1 being fully methylated. At each CpG site, methylation is quantified by [β = M/(M + U + α)], where M > 0 and U > 0 denote the methylated and unmethylated signal intensities, respectively; and an offset, α, (equal to 1 by default) [35]. TCGA: β-values (Array-based DNA platform) were used to determine methylation levels of *LRIG1*, in adjacent normal versus breast tumour samples, and within breast tumour molecular subtypes. Welch two-sample *t* test, or ANOVA followed by a Tukey HSD test, was used to determine whether the means of the two groups, or three or more groups, respectively, were statistically different. The relationship between *LRIG1* methylation and *LRIG1* mRNA expression for breast tumour samples was determined using β-values and mRNASeq read count data. Methylation values and mRNA expression values were matched by patient sample, and correlation was computed using the Spearman rank correlation coefficient method. Molecular subtyping was based on the PAM50 (Prosigna Breast Cancer Prognostic Gene Signature Assay) profiling test. Kaplan–Meier survival plots were generated using the ‘survival’ and ‘survminer’ packages in the R statistical program. Data for Kaplan–Meier plots consisted of β-values and patient clinical information (death status and days to last contact) and used data-driven cut-offs computed by survminer. A difference in survival probability between groups was determined using the Tarone-Ware test in survminer. GSE78758: β-values (Illumina HumanMethylation450 BeadChip) were used to determine methylation levels of *LRIG1* in adjacent normal, primary breast tumour, and lymph node metastases in basal/triple-negative breast cancer patients. A one-way ANOVA followed by a post ad hoc Tukey HSD test, was used to determine whether the means of the three groups were statistically different.

### Statistical analysis

Statistical tests for data analysis included Welch two-sample *t* test, ANOVA followed by Tukey’s HSD test, log-rank test, and Student’s *t* test. Values are represented as mean ± standard error of the mean (SEM). For in vitro experiments, data represent at least three independent experiments (unless otherwise specified in the figure legend). In all cases, differences were considered statistically significant when *P* value was less than 0.05. All graphical representation and statistical analyses of data were performed using the R statistical programme or Microsoft Excel.

## Results

### *LRIG1* methylation is increased in breast cancer

Prior studies in colorectal and cervical cancers indicate that the *LRIG1* locus is hypermethylated [22, 23]. Interestingly, a recent study using canine mammary tumours as a model for human breast cancer, identified hypermethylation in the third intron of *LRIG1* overlapping with a tumour suppressive PAX5 DNA binding motif. Hypermethylation at this region correlated with reduced *LRIG1* gene expression, which was conserved (along with PAX5-binding motifs) in human breast cancer [36], suggesting that methylation of *LRIG1* in cancer may be a species-conserved mechanism of repression. Here, we sought to explore methylation at the *LRIG1* CpG island, versus intronic regions, as hypermethylation of tumour suppressor genes in the CpG islands near the promoter is a well-documented feature of cancer.

**Fig. 1:**
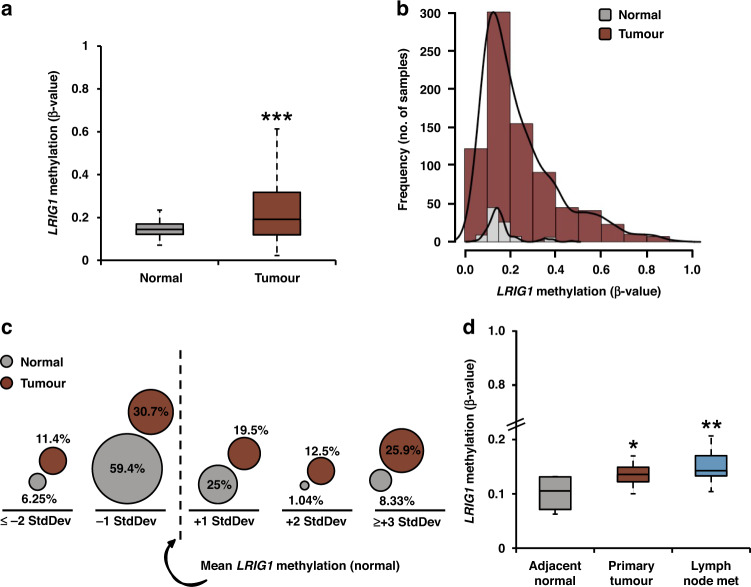
*LRIG1* methylation in human breast cancer. **a** Boxplot and (**b**) histograms (with overlying density plots) depicting *LRIG1* β-values in normal breast tissue (*n* = 96) and breast tumours (*n* = 796) (data from TCGA BRCA). **c** Dot plot depicting the percent distribution of *LRIG1* β-values in normal breast tissue and breast tumour samples relative to the *LRIG1* β-value mean in normal samples (vertical dotted line) (data from TCGA BRCA). **d** Boxplot depicting *LRIG1* β-values in adjacent normal breast tissue (*n* = 4), primary tumours (*n* = 23) and lymph node metastases (*n* = 12) from patients with triple-negative breast cancer (data from GSE78758). *P < 0.05, **P < 0.01 and ***P < 0.001. Students *t* test (**a**), Tukey’s HSD (**d**).

Supplemental Fig. 1 depicts the location of the promoter-proximal CpG island, relative to the transcription start site and exon 1 of the *LRIG1* gene. To examine whether *LRIG1* is methylated in breast cancer, we utilised the publicly available TCGA-BRCA dataset, useful for both its large sample size and matched patient methylation-mRNA expression data. (Beta values (β-values) are the estimate of methylation at a given CpG site. β-values range from 0 to 1.0, with 0 being fully unmethylated and 1.0 being fully methylated; and are quantified by β = M/(M + U), where M and U denote methylated and unmethylated signal intensities, respectively [35]). Analysis of methylation status using the HumanMethylation450 array revealed that methylation (β-value) at the *LRIG1* CpG island is significantly increased in breast tumours (*n* = 796) compared to normal samples (*n* = 96) (Fig. 1a, b). Relative to the mean methylation value in normal samples, tumour samples are more highly represented at values that are 2 or ≥3 standard deviations above this normal mean (Fig. 1c). Furthermore, methylation of *LRIG1* is increased in primary tumours and lymph node metastases of patients with basal breast cancer/TNBC relative to normal tissue (Fig. 1d, data from GSE78758 [37]), suggesting methylation-mediated silencing of *LRIG1* may be important in breast cancer progression.

### *LRIG1* methylation inversely correlates with LRIG1 expression

**Fig. 2:**
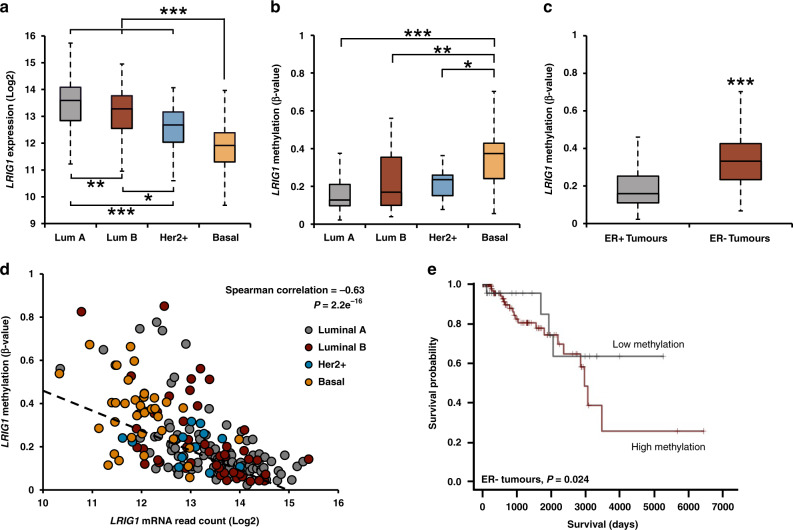
*LRIG1* DNA methylation negatively regulates *LRIG1* mRNA expression. **a** Boxplot depicting *LRIG1* mRNA expression (read count) in Luminal A (*n* = 439), Luminal B (*n* = 118), Her2 + (*n* = 37) and Basal (*n* = 112) breast tumours (data from TCGA). **b** Boxplot depicting LRIG1 β-values in Luminal A (*n* = 110), Luminal B (*n* = 46), Her2 + (*n* = 14) and Basal (*n* = 41) breast tumours (data from TCGA). **c** Boxplot depicting *LRIG1* β-values in ER-positive (*n* = 565) and ER-negative (*n* = 167) breast tumours (data from TCGA BRCA). **d** Scatter plot of patient-matched *LRIG1* mRNA read count and *LRIG1* β-values in breast tumour samples (*n* = 204). Black dotted line indicates linear regression (data from TCGA BRCA). **e** Kaplan–Meier plot of overall survival as a function *LRIG1* methylation in ER-negative tumours. Patients were segregated into groups based on ERα status and *LRIG1* β-value in their primary tumour (high methylation/ER-negative: *n* = 135, low methylation/ER-negative: *n* = 32) (data from TCGA BRCA). *P < 0.05, **P < 0.01 and ***P < 0.001, respectively; Tukey’s HSD (**a**, **b**), Student’s *t* test (**c**), Spearman correlation test (**d**), log-rank test (**e**).

We previously reported that *LRIG1* expression is lowest in the basal-like molecular subtype of breast cancer [19]. To determine if this pattern also exists in the larger TCGA-BRCA dataset, we examined *LRIG1* expression as a function of breast cancer molecular subtypes. Analysis of mRNA read count (RNASeqV2) confirmed that *LRIG1* is most significantly reduced in basal breast tumours (Fig. 2a). We, therefore, queried the *LRIG1* CpG island methylation status as a function of molecular subtype, as shown in Fig. 2b. Interestingly, CpG island methylation was significantly higher in basal breast tumours, as compared to the Luminal A, Luminal B and Her2+ subtypes. When tumours were segregated based on ERα status, ER-negative tumours (which encompass basal and most Her2+ breast cancers) demonstrated increased methylation (Fig. 2c), in agreement with our prior findings that *LRIG1* is poorly expressed in Her2+ and basal breast tumours [19, 38], while richly expressed in ER-positive tumours [14]. We next examined the correlation between *LRIG1* CpG island methylation and *LRIG1* mRNA read count in breast cancer (*n* = 784). As shown in Fig. 2d, there is a striking inverse correlation between methylation and mRNA expression (Spearman correlation −0.63, *P* < 0.001), strongly suggesting that methylation decreases *LRIG1* expression. (Supplemental Table 1 shows correlation by molecular subtype.) Given that ER-negative tumours show the most robust methylation, we performed Kaplan–Meier analysis on patients with ER-negative tumours to assess whether there was a correlation between *LRIG1* CpG island methylation status and overall patient survival. Patients whose breast tumours expressed higher levels of *LRIG1* methylation had decreased overall survival time (Fig. 2e), suggesting that *LRIG1* promoter methylation is functionally important.

Recent studies have revealed that methylation of distal-regulatory regions, including enhancers, plays an important role in regulating gene expression [39, 40]. In prior work, we demonstrated that *LRIG1* is an ER regulated gene [14]. Interestingly, Stone et al. reported that enhancer hypermethylation within ERα regulated genes is correlated both with decreased ERα binding and decreased gene expression [41]. Mining of supplemental data provided by Stone et al. revealed two ERα binding sites within *LRIG1* enhancer regions which are hypermethylated (HumanMethylation450 CpG probes cg24150385 and cg09716921, herein labelled eCpG-1 and eCpG-2, respectively). We were curious to determine whether these elements display subtype-specific methylation, as observed for the CpG island. Indeed, as shown in Supplemental Fig. 2A, methylation of both eCpG-1 and eCpG-2 is highest in the basal subtype of breast cancer. Methylation of both elements inversely correlates with *LRIG1* gene expression, in the TCGA-BRCA cohort (Supplemental Fig. 2B) and in multiple cell line models of endocrine-resistant breast cancer [41]. (Supplemental Table 1 shows correlation by molecular subtype.) Kaplan–Meier analysis of ER-negative patients revealed that patients whose breast tumours expressed higher levels of *LRIG1* methylation, at these enhancer-region CpG sites, had decreased overall survival time (Supplemental Fig. 2C), suggesting that methylation at both elements is functional. Collectively, these findings suggest that loss of *LRIG1* expression in basal/TNBC is multifactorial, with contributions from both promoter and enhancer methylation.

### *LRIG1* methylation in breast cancer cell lines

**Fig. 3:**
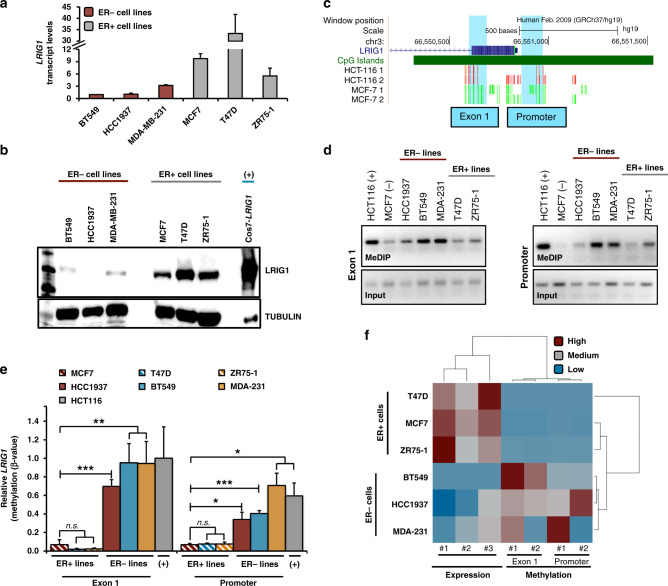
*LRIG1* methylation in breast cancer cell lines. **a** Bar plot showing relative *LRIG1* transcript levels across breast cancer cell lines (*n* = 6). Expression was determined from the Ct values using the 2^−ΔΔCt^ method after normalisation to *GAPDH*. *LRIG1* transcript levels were normalised to BT549 values, which are set to 1.0. **b** LRIG1 protein abundance across breast cancer cell lines (*n* = 6), and Cos7 cells with stable overexpression of LRIG1, assessed by western blotting. Tubulin serves as a loading control. **c** Snapshot of *LRIG1* gene and promoter-proximal CpG island in the UCSC Genome Browser hg19 build. DNA Methylation by Reduced Representation Bisulfite-Sequencing (RRBS) track depicts the location and percent methylation of CpG sites in HCT116 and MCF7 cells. Red: 100% methylation, yellow: 50% methylation, green: 0% methylation. Exon 1 and Promoter regions are highlighted. **d** DNA agarose gels of MeDIP-PCR show methylation levels at Exon 1 and Promoter regions across cell lines (*n* = 7). In all, 5% of input DNA is shown in the bottom panels. **e** Bar plot showing relative β-values at LRIG1 Exon 1 and Promoter regions as assayed by MeDIP-qPCR. The methylation level was determined from the Ct values using the 2^−ΔΔCt^ method after normalisation to 5% input DNA. β-value was determined by normalisation to HCT116 Exon 1 methylation value, which is set to 1.0. **f** Heatmap of *LRIG1* levels across replicates of transcript expression (data from (**a**)) compared to Exon 1 and Promoter region methylation levels (data from (**e**)), respectively, in breast cancer cell lines (*n* = 6). Values are mean ± SEM of two (**d**, **e**) or three independent experiments. *P < 0.05, **P < 0.01 and ***P < 0.001. n.s. not significant (*P* > 0.05); Student’s *t* test (**e**).

Having examined publicly available patient samples, we next examined *LRIG1* CpG island methylation in breast cancer cell line models. We chose several cell lines, including MCF7 and T47D (Luminal A) and ZR75-1 (Luminal B), representative of ER-positive breast cancer, and BT549, HCC1937 (BRCA1 mutant) and MDA-MB-231, representative of basal/TNBC. *LRIG1* mRNA transcript expression (Fig. 3a) and protein abundance (Fig. 3b) were comparably higher in the ER-positive breast cancer cell lines, as expected [14, 19]. To focus our analysis, we used the UCSC genome browser to query methylation levels across the *LRIG1* CpG island (Fig. 3c) [26]. HCT116 colon carcinoma cells were included as a positive control, as they are reported to have high levels of *LRIG1* CpG island methylation [22]. Notably, regions of the CpG island are highly methylated (red bars; 100% methylation) in HCT116 cells, while these same regions are unmethylated (green bars; 0% methylation) in MCF7 breast cancer cells. Using this information, we focused our analysis on two regions that exhibited differential methylation between HCT116 and MCF7 cell lines, designated “Exon 1” and “Promoter” in Fig. 3c. Using the methylated DNA immunoprecipitation (MeDIP) technique, which immunoprecipitates methylated DNA using an antibody specific for 5-methylcytosine [42], we assayed methylation levels across human breast cancer cell lines. A representative agarose gel (Fig. 3d) and relative methylation levels (Fig. 3e), based on qPCR, are shown for both regions. The promoter region of the small nuclear ribonucleoprotein polypeptide N gene (*SNRPN/SNURF*), which is heavily methylated [43], served as a control for the MeDIP technique and illustrates that differential methylation observed at the *LRIG1* CpG island is not due to a global increase or decrease in methylation inherent to each cell line (Supplemental Fig. 3A, B). We found that methylation levels at the Exon 1 and Promoter regions of *LRIG1* inversely correlate with *LRIG1* mRNA expression levels (Fig. 3f). Interestingly, the results in the breast cancer cell lines reflected our findings in patient samples, with methylation lowest in ER-positive breast cancer lines and highest in basal/triple-negative cell lines (ER-negative). This suggests that *LRIG1* silencing by methylation may be an inherent property of basal/TNBC.

### Inhibition of DNA methylation induces *LRIG1* mRNA expression

**Fig. 4:**
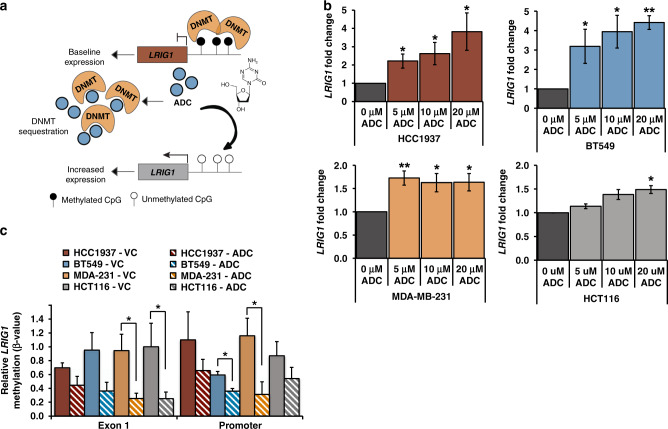
Inhibiting DNA methylation rescues *LRIG1* mRNA expression. **a** Schematic representation of *LRIG1* demethylation following ADC (5-Aza-2’-deoxycytidine) treatment. ADC (nucleotide structure, inset) sequesters DNA methyltransferases (DNMTs) resulting in demethylation of *LRIG1* DNA and transcriptional activation. **b** Bar plots showing relative *LRIG1* expression levels in ER-negative breast cancer cell lines (BT549, HCC1937, MDA-MB-231) and colon cancer cell line (HCT116) treated with shown concentrations of ADC for 96 h. Expression was determined from the Ct values using the 2^−ΔΔCt^ method after normalisation to *GAPDH*. *LRIG1* fold changes were normalised to 0 μM treatments, which are set to 1.0. **c** Bar plot showing relative β-values at *LRIG1* Exon 1 and Promoter regions, as assayed by MeDIP-qPCR, in cells treated with vehicle control (VC) or 20 μM ADC for 96 h. Methylation level was determined from the Ct values using the 2^−ΔΔCt^ method after normalisation to 5% input DNA. β-value was determined by normalisation to HCT116 Exon 1 VC methylation value, which is set to 1.0. Values are mean ± SEM of three (**b**) or two (**c**) independent experiments. *P < 0.05 and **P < 0.01. Student’s *t* test (**b**, **c**).

We next determined the impact of the DNA methyltransferase inhibitor, 5-aza-2’-deoxycytidine (ADC), on *LRIG1* mRNA expression. As shown in Fig. 4a, ADC sequesters DNA methyltransferases resulting in the demethylation of *LRIG1* DNA and subsequent transcriptional activation. ADC treatment consistently increased *LRIG1* mRNA expression in the basal/TNBC cell lines (BT549, HCC1937, and MDA-MB-231), and in heavily methylated HCT116 cells at high doses (Fig. 4b). Interestingly, ADC treatment of ER-positive breast cancer cell lines (T47D, MCF7, and ZR75-1) either significantly decreased, or had little/no effect, on *LRIG1* expression (Supplemental Fig. 4a). To further examine ADC-induced methylation inhibition, we used MeDIP to measure methylation levels at the *LRIG1* Exon 1 and Promoter regions (from Fig. 3c), following ADC treatment. Indeed, we found that relative *LRIG1* methylation levels were significantly decreased at both regions in cells treated with ADC versus vehicle control (Fig. 4c).

Since histone deacetylases (HDACs) repress gene expression by compacting chromatin structure and can be recruited to methylated DNA by methyl-binding proteins [44], we investigated whether HDACs could also be contributing to the silencing of *LRIG1* in basal/TNBC. Treatment of cells with the HDAC inhibitor, Panobinostat, as a single agent did not significantly increase *LRIG1* mRNA expression (Supplemental Fig. 4B). We next went on to test the combination of Panobinostat and ADC, finding this was not consistently superior to ADC alone (except in HCC1937 cells); suggesting that methylation, not deacetylation, is the dominant epigenetic mechanism of *LRIG1* silencing in breast cancer.

### The CRISPR-deadCas9 transactivation system reactivates *LRIG1*

**Fig. 5:**
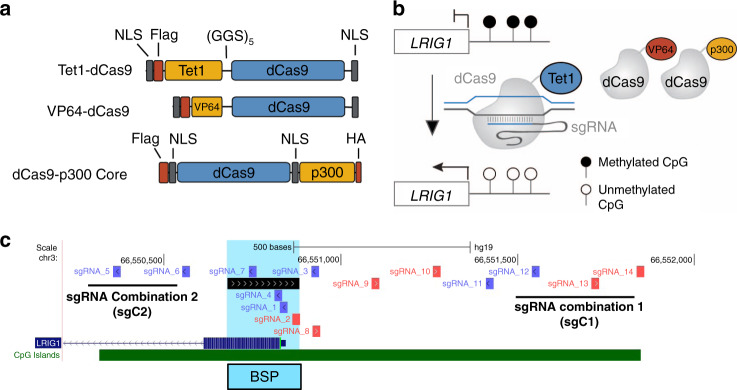
Targeting *LRIG1* using the dCas9 transactivation system. **a** Schematic representation of construction of dCas9 plasmids. The resulting plasmids contain dCas9 in direct fusion with Tet1, VP64 or p300 (Core). **b** Schematic representation of the sgRNA-Tet1-dCas9 complex. dCas9 is in direct C-terminal fusion with the demethylation-initiating enzyme Tet1 or the transcriptional activators VP64 or p300 (Core). sgRNAs bind in CpG island regions of *LRIG1* to activate gene expression. **c** Snapshot of sgRNA target sites in the *LRIG1* CpG island region. Arrowheads indicate whether the sgRNA targets the forward or reverse DNA strand. Blue arrowheads indicate “+” DNA strand. Red arrowheads indicate “−” DNA strand. Bisulfite-sequencing primer (BSP) region is highlighted. Guide RNA combinations are underlined. sgC1; sgRNA combination 1 includes sgRNA 12, sgRNA 13, and sgRNA 14. sgC2; sgRNA combination 2 includes sgRNA 5 and sgRNA 6.

LRIG1 mRNA and protein are consistently decreased across cancer types, including breast cancer. However, genetic alterations in *LRIG1*, such as copy number variation, are observed in less than 1% of breast cancers (Supplemental Fig. 5), indicating that epigenetic silencing is a dominant mechanism of *LRIG1* loss. This suggests that *LRIG1* may be amenable to “transcriptional reactivation”, which is now feasible using targeted approaches. Towards this end, we employed the CRISPR/deadCas9 transactivation system, which utilises a catalytically inactive Cas9 protein (dCas9) directly fused to either the: (1) ten-eleven translocation methylcytosine dioxygenase 1 (TET1) enzyme (Tet1-dCas9), (2) VP64 domain (VP64-dCas9), or (3) catalytic core of acetyltransferase p300 (dCas9-p300 Core) (Fig. 5a, b). TET enzymes accomplish demethylation by catalysing the conversion of 5-methylcytosine (5mC) to 5-hydroxymethylcytosine (5hmC), and further to 5-formylcytosine (5fC) and 5-carboxylcytosine (5caC). 5fC and 5caC are removed by cellular DNA repair machinery, regenerating unmethylated cytosines [45, 46]. VP64 is composed of four tandem copies of VP16 (Herpes Simplex Viral Protein 16) connected by glycine–serine linkers and acts as a strong transcriptional activator through recruitment of transcription factors (e.g., TATA-binding protein (TBP) and TBP-related factors) and recruitment of chromatin modification factors (e.g., histone-modification factors) [47]. The transcriptional co-activator p300 is a histone acetyltransferase that regulates gene expression by catalysing acetylation of histone H3 lysine 27 (H3K27) and is typically recruited to promoter and enhancer regions of target genes [29]. We designed 14 sgRNAs (Fig. 5c) spanning the *LRIG1* CpG island within a ± 1 kb window on either side of the *LR1G1* transcriptional start site (TSS) and evaluated the activation ability of dCas9 fusions.

**Fig. 6:**
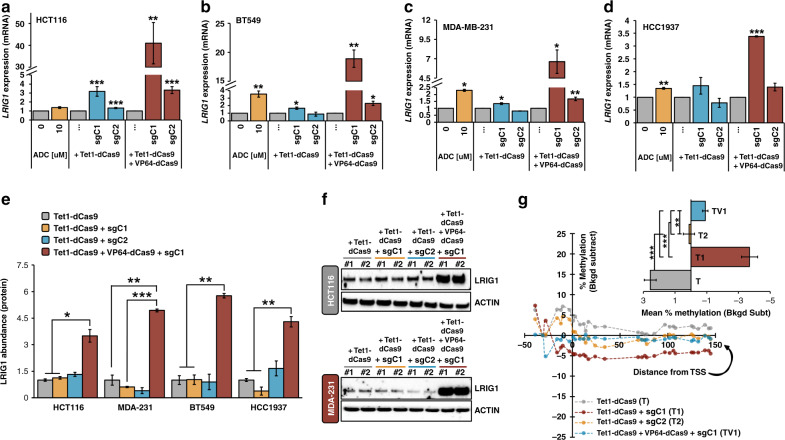
*LRIG1* is activated by the dCas9 transactivation system in TNBC cells. **a**–**d** Bar plots showing relative *LRIG1* expression levels in HCT116 (**a**), MDA-MB-231 (**b**), BT549 (**c**) and HCC1937 (**d**) cells treated with 0μM (vehicle control) or 10 μM ADC, transfected with Tet1-dCas9 alone or with guide RNA combinations (sgC1, sgC2), or transfected with a combination of Tet1-dCas9 and VP64-dCas9 alone or with guide RNA combinations (sgC1, sgC2). Expression was determined from the Ct values using the 2^−ΔΔCt^ method after normalisation to *GAPDH. LRIG1* fold changes were normalised to 0 μM ADC or no sgRNA values for each group, which are set to 1.0. **e** Quantification of LRIG1 protein abundance in cell lines (*n* = 4) transfected with Tet1-dCas9 alone, Tet1-dCas9 with sgC1 or sgC2 and Tet1-dCas9 and VP64-dCas9 with sgC1 as assessed by Western Blotting. Actin serves as a loading control. Protein abundance was determined by densitometry using Image J after normalising to Tet1-dCas9 values, which are set to 1.0. **f** Images of HCT116 and MDA-MB-231 blots are shown. **g** Line plot of change in percent methylation in HCT116 cells transfected with Tet1-dCas9, Tet1-dCas9 with guide RNA combinations (sgC1, sgC2), or a combination of Tet1-dCas9 and VP64-dCas9 with sgC1 as assessed by targeted sequencing of bisulfite converted genomic DNA across 30 CpG dinucleotides near the *LRIG1* transcriptional start site. Change in percent methylation was determined after background subtracting the no treatment control percent methylation values. The *X* axis depicts CpG distances from *LRIG1* transcriptional start site (at 0). Values are mean ± SEM of two (**a**–**d**, **g**) or three (**e**, **f**) independent experiments. *P < 0.05, **P < 0.01 and ***P < 0.001. Student’s *t* test (**a**–**e**, **g**).

The dCas9 transactivation system was expressed in cell lines by lipid-based transfection followed by antibiotic selection 24 h post-transfection (HPT) to enrich for transfected cells (Supplemental Fig. 6A). As previous studies using CRISPR/dCas9 to reactivate gene expression have shown that combined delivery of multiple sgRNAs provides increased levels of endogenous gene activation [48–50], we tested several combinations of 2–3 guide RNAs (Supplemental Fig. 6B). We grouped guides based on their genomic locations across the *LRIG1* CpG island. We performed RT-qPCR and observed significant activation of *LRIG1* expression with the combination of sgRNAs 12–14 (termed sgC1) and sgRNA 5,6 (termed sgC2) paired with Tet1-dCas9, targeting regions both up- and downstream of the TSS. *LRIG1* expression increased 1.3 to 3.1-fold in HCT116 cells relative to Tet1-dCas9 with no guide RNA (Fig. 6a). No significant difference was observed between cells transfected with Tet1-dCas9 alone and Tet1-dCas9 coupled with sgRNAs 9–11, 2,3,8 or 1,4,7 (Supplemental Fig. 6B). In concordance with HCT116 cells, transfection with Tet1-dCas9 and sgC1 revealed a significant upregulation of *LRIG1* in BT549 (1.7-fold, Fig. 6b) and MDA-MB-231 (1.3-fold, Fig. 6c) cells when compared to cells transfected with Tet1-dCas9. (HCC1937 cells showed a 1.4-fold upregulation, but was not statistically significant, Fig. 6d). No significant upregulation was observed in basal/TNBC cell lines transfected with Tet1-dCas9 with sgC2 versus no sgRNAs. Cells treated with 10 μM ADC were included as a positive control, as we previously showed ADC treatment consistently increases *LRIG1* expression. We also tested Tet1-dCas9 paired with randomised combinations of *LRIG1* targeting sgRNAs and still observed significant increases in *LRIG1* transcript expression (Supplemental Fig. 6C), suggesting that *LRIG1* upregulation can be achieved using multiple varying combinations of guide RNAs.

As others have previously observed enhanced activation when co-targeting dCas9 complexes [49], we were therefore curious whether other epigenetic mechanisms, such as transcriptional activation, could also upregulate *LRIG1* transcript expression. To this end, we transfected cells with VP64-dCas9 paired with sgC1 or sgC2 guide RNA combinations alone (Supplemental Fig. 6D, E) or in combination with Tet1-dCas9 (Fig. 6a–d). We observed a 3.4 to 18.9-fold upregulation of *LRIG1* in basal/TNBC cells (41-fold in HCT116 cells) for sgC1, and a 1.4 to 2.3-fold increase for sgC2 (3.3-fold in HCT116 cells) when compared to cells transfected with Tet1-dCas9/VP64-dCas9 with no guides. We also tested dCas9-p300 (Core) paired with sgC1 or sgC2 guide RNA combinations alone (Supplemental Figs. 6D, E) or in combination with Tet1-dCas9 (HCT116 only, Supplemental Fig. 6E), observing no significant upregulation of *LRIG1* transcript expression. Moreover, in HCT116 cells a triple combination of Tet1-dCas9, VP64-dCas9, and dCas9-p300 with sgC1 or sgC2, although significant, showed lower levels of *LRIG1* expression (Supplemental Fig. 6E). As p300 regulates gene expression through acetylation, this data suggests that deacetylation does not contribute to *LRIG1* silencing in line with our data from HDAC treatment in Supplemental Fig. 4B.

To investigate the possibility of off-target gene regulation, we individually analysed sgRNAs 12–14 (sgC1) and 5–6 (sgC2) sequences to identify putative genome-wide off-target sgRNA-binding sites. We then selected genes which were identified as off-targets for at least two sgRNAs in Combination 1 and for both sgRNAs in Combination 2 (Supplemental Fig. 7). RT-qPCR was performed to assess the five target genes in HCT116 cells transfected with Tet1-dCas9 or Tet1-dCas9/VP64-dCas9 with sgC1 or sgC2. We observed no significant upregulation on the expression of the putative off-target genes compared to no sgRNA control conditions (Supplemental Fig. 6F), supporting site-specific regulation of *LRIG1* by dCas9 protein complexes.

### The dCas9 system activates LRIG1 protein expression and inhibits cell viability

To determine whether activation of *LRIG1* by Tet1-dCas9 and VP64-dCas9 extends beyond upregulation of transcript expression, we assessed LRIG1 protein levels and cell viability in HCT116 and basal/TNBC cells. For these experiments, we focused much of our attention on sgC1 as it caused higher levels of *LRIG1* upregulation compared to sgC2. Only cells transfected with the combination of Tet1-dCas9 and VP64-dCas9 with sgC1 showed a significant increase (3.5 to 5.8-fold) in LRIG1 protein abundance (Fig. 6e, f and Supplemental Fig. 8A). These data may suggest that demethylation alone is not sufficient to increase LRIG1 protein abundance, and that gene-specific mRNA expression thresholds must be reached to observe concomitant protein upregulation.

We next examined the functional impact of reactivation of LRIG1 on the viability of cancer cells (Supplemental Fig. 8B). Treatment with 10 μM ADC was included as readout of global demethylation-induced changes in cell viability, and we observed significant decreases in cell viability following ADC treatment. Tet1-dCas9 and Tet1-dCas9/VP64-dCas9, both paired with sgC1, significantly decreased the viability of HCT116 cells and all three basal/TNBC cell lines when compared to no guide RNA conditions. Notably, we found that both Tet1-dCas9 with sgC1 and Tet1-dCas9/VP64-dCas9 with sgC1 were more effective at reducing cell viability compared to ADC treatment. These data suggest that targeted reactivation of *LRIG1* provides superior effects compared to a global demethylation agent. This observed difference could be due to the complex crosstalk between a large number of methylated genes in the human genome, as well as their likely unequal contributions towards regulating cancer cell viability.

### Tet1-dCas9 significantly reduces DNA methylation

Lastly, due to differences in reactivation of *LRIG1* between the dCas9 effector domains and sgRNA combinations, we quantitatively determined the extent of demethylation in the core promoter region of *LRIG1* using targeted bisulfite amplicon sequencing. We generated PCR-based amplicons which allowed us to measure percent methylation (ratio of 5-meCG/total CG) across 30 individual CpG dinucleotides in the selected region (185 base pairs, Fig. 5c) by deep sequencing. We observed significant decreases in DNA methylation across this region, starting 16–28 base pairs upstream of the *LRIG1* TSS, following transfection of dCas9/sgRNA complexes. Specifically, in HCT116 cells, mean methylation decreased by 6.3%, 2.4% and 3.5% in cells transfected with Tet1-dCas9 with sgC1, Tet1-dCas9 with sgC2, and Tet1-dCas9/VP64-dCas9 with sgC1, respectively, compared to no guide RNA control (Fig. 6g, g inset). BT549 cells transfected with Tet1-dCas9 with either sgC1 or sgC2 exhibited significant decreases in methylation (Supplemental Fig. 8C, C inset). HCC1937 cells also displayed uniform decreases in methylation across experimental conditions versus Tet1-dCas9 only controls (Supplemental Fig. 8D, D inset). We observed minimal, non-significant decreases in methylation levels in MDA-MB-231 cells (Supplemental Fig. 8E). (Supplemental Fig. 8f shows methylation levels in untreated cells which were used for background subtraction calculations.) Interestingly, in HCT116, BT549, and HCC1937 cells, although the addition of VP64-dCas9 significantly reduced methylation levels compared to control, it did not further reduce percent methylation compared to Tet1-dCas9 with sgC1. In fact, in HCT116 and BT549 cells, percent methylation increased between the Tet1-dCas9 with sgC1 and Tet1-dCas9/VP64-dCas9 with sgC1 conditions. This observation may indicate that TET1 protein needs to achieve a minimum threshold of demethylation that subsequently permits transcriptional activation of *LRIG1* by VP64. In addition, these data suggest in line with prior studies [49], that VP64-dCas9 does not use demethylation as a means towards transcriptional activation.

## Discussion

*LRIG1* is a negative regulator of oncogenic receptor tyrosine kinases and a documented tumour suppressor [51]. Indeed, *LRIG1* downregulation has prognostic impact across diverse tumour types [52]. *LRIG1* downregulation in cancer is widespread, with a recent study reporting “no to low” *LRIG1* expression in cell lines from 22 different cancer types, and patient samples from seventeen different cancer types [53]. Analysis of a genome-wide RNAi screen [54] found that *LRIG1* is one of six genes which rank in the top 1% (out of 17,080 genes) whose knockdown promotes cancer cell proliferation, across 43 different cancer cell lines [53]. Given these findings, much discussion has focused on how to “harness” LRIG1 for potential therapeutic benefit [55]. Several studies have reported that delivery of the soluble extracellular domain of LRIG1 holds promise [56, 57], although further work is needed to define the key functional domains of LRIG1. An alternative approach is to restore endogenous *LRIG1* expression, although this requires greater insight into mechanisms that silence *LRIG1* in cancer.

Our study focused on *LRIG1* silencing in breast cancer. As *LRIG1* copy number variations are rare in cancer, we hypothesised that epigenetic silencing of *LRIG1* would be prevalent and explain its more significant loss in ER-negative and basal/TNBC disease. Indeed, we find that methylation of the *LRIG1* CpG island and ERα-bound enhancers is significantly increased in ER-negative and basal/TNBCs and that methylation inversely correlates with *LRIG1* mRNA expression. We demonstrate that subtype-specific differential methylation is observed in cell line models of ER-positive and ER-negative breast cancer and inversely correlate with LRIG1 expression in these cell lines. Furthermore, we show that robust *LRIG1* methylation correlates with poor overall survival in patients with ER-negative breast tumours.

Global hypomethylating agents such as 5-aza-2’-deoxycytidine (ADC, Decitabine) are approved for the treatment of myelodysplastic syndromes and have been in use in that setting for more than a decade [58]. We used ADC, a DNA methyltransferase inhibitor, as an experimental tool to examine whether *LRIG1* expression could be induced in breast cancer cells by CpG island demethylation. Indeed, ADC treatment of ER-negative/TNBC cells significantly increased *LRIG1* mRNA expression. In contrast, ADC had no significant effect on *LRIG1* expression in ER-positive cells, suggesting that low-level methylation observed in this setting is not functionally important. Since ADC-induced *LRIG1* upregulation may occur indirectly, using methylated DNA immunoprecipitation (MeDIP) we demonstrate that demethylation occurs at the CpG island following ADC treatment. Due to its ability to globally reduce methylation levels, ADC has been implicated as a potential cancer therapeutic, with the ability to reduce cancer cell proliferation and migration and induce apoptosis [59–61]. In this study, we observe that ADC treatment reduces the viability of colorectal carcinoma and TNBC cells, and although high ADC concentrations (≥20 μM) likely induce apoptosis in a portion of the cell population, there is no evidence or expectation that this should impact upregulation of *LRIG1*.

Tumour suppressor restoration is an active area of study and holds great promise. Due to advances in CRISPR/Cas9-based technology, targeted transcriptional reactivation of tumour suppressor genes is now feasible. In fact, this has been demonstrated for PTEN and BRCA1, using dCas9 fused to the VPR effector domain (a potent transactivator consisting of VP64, p65 and Rta) and TET1; respectively [50, 62]. Genome-based CRISPR/dCas9 activation allows precise and stable editing of the epigenome with high efficiency, which could be harnessed for precision medicine and treatment of LRIG1-defincient cancers. In this study, we focused specifically on the role of the CpG island methylation in silencing *LRIG1*. Using Tet1-dCas9, we demonstrate for the first time, that targeted demethylation of the promoter-proximal CpG island induces *LRIG1* expression in breast cancer cells. We observed that sgRNA combinations designed against binding sites furthest from the *LRIG1* transcriptional starts site were most effective, suggesting that both the location of guide RNAs relative to transcriptional start and CpG sites, as well as the chosen combination, may affect the efficiency of TET1 activity. *LRIG1* reactivation, both at the mRNA and protein levels, showed significant, but variable, responses across cell lines. This is consistent with previous studies using dCas9-based epigenetic editing in which target gene responses varied by cell type and genomic locus [28, 63]. However, we did observe ~1.3- to threefold upregulation of *LRIG1* expression with Tet1-dCas9 which is consistent with the ~1.5 to 2.3-fold increase in target genes also reactivated via CRISPR/dCas9/TET1 demethylation [49, 62]. In addition to cell type and DNA location, variable responses may also be attributed to differences in other epigenetic modifiers of *LRIG1*. For example, previous studies have shown that *LRIG1* is regulated by histone deacetylation [64], microRNAs [65, 66] and long noncoding RNAs [67]; all of which may contribute to the level of *LRIG1* reactivation. This may result in variable levels of baseline methylation even amongst ER-negative cell lines (as observed in Fig. 3e). As the CRISPR/dCas9 system was delivered to cells via lipid-based transient transfection, we acknowledge that transfection efficiency may also contribute to observed differences in *LRIG1* reactivation. However, since prior studies have shown global methylation changes and higher off-target effects with stably expressed dCas9 fusions to TET1 and DNA methyltransferase DNMT3A [49, 68, 69], we opted for transient expression, attempting to minimise these caveats. Our dCas9 transactivation system also showed specificity towards targeting *LRIG1*, with no observed upregulation of the five off-targeted tested.

Due to the strong effects of VP64 and p300 on gene upregulation, we sought to assess if these effectors could contribute to *LRIG1* reactivation via CRISPR/dCas9. We found that the combinatorial binding of VP64-dCas9 and Tet1-dCas9 was capable of significantly upregulating *LRIG1* mRNA expression and protein abundance. However, unexpectedly, these effects were not synergistic but were largely due to VP64-induced transcriptional activation as transfection with VP64-dCas9 and sgRNAs alone exhibited slightly higher *LRIG1* expression compared to the Tet1-dCas9/VP64-dCas9 combination. This could be due to steric hinderance caused by large dCas9 protein complexes attempting to bind in the same genomic region [70, 71], especially regions with high binding affinity, such as CpG islands, promoters, and enhancers. However, we cannot rule out the possibilities that: (1) demethylation by the presence of dCas9 alone or TET1 alone, which has been shown in prior studies [69, 72], leads to a chromatin state more permissive of VP64-induced transcriptional activation, or (2) strong and targeted transcriptional activation by VP64 may be able to overcome or circumvent methylation-based gene silencing. Indeed, bisulfite modified sequencing shows that gene upregulation by VP64 is not dependent on demethylation.

To validate its biological relevance, we assessed the effects of Tet1/VP64-dCas9-mediated *LRIG1* expression on cell viability. The significant reduction in cancer cell viability suggests that *LRIG1* reactivation via CRISPR/dCas9 may have an impact on TNBC growth, however, future studies will be required to assess biological relevance in more disease-relevant models such as patient-derived organoids and in vivo mouse models. It is unclear how long demethylation and transcriptional activation by Tet1-dCas9 and VP64-dCas9, respectively, would be maintained in vivo, as well as maintenance of downstream effects. In addition, future studies, using readily available single-cell sequencing technologies, will be required to determine whether the observed reactivation of *LRIG1*, and other target genes, is due to uniform reactivation at the population level or strong reactivation in only a subset of the cell population. Collectively, our data provide strong evidence that *LRIG1* is silenced by both CpG island and enhancer methylation in TNBC, and that the CRISPR/dCas9 system can be used to upregulate *LRIG1* expression. While this study has some limitations, it provides the first evidence that a targeted approach using site-specific demethylation and transcriptional activation, is a feasible method of restoring *LRIG1*. As *LRIG1* is known to play a crucial role in tumorigenesis and metastasis, combining CRISPR/dCas9-based reactivation with conventional therapeutic approaches could hold promise for *LRIG1*-silenced tumours.

## Supplementary information

Supplemental Figure 1

Supplemental Figure 2

Supplemental Figure 3

Supplemental Figure 4

Supplemental Figure 5

Supplemental Figure 6

Supplemental Figure 7

Supplemental Figure 8

Supplemental Figure 9

Supplemental Table 1

Supplemental Table 2

Supplemental Figure Legends

## Data Availability

The datasets analysed in this study are available from the NCBI Gene Expression Omnibus (GEO) data repository (https://www.ncbi.nlm.nih.gov/geo) or The Cancer Genome Atlas Breast Invasive Carcinoma data repositories (https://portal.gdc.cancer.gov/projects/TCGA-BRCA).
