# Supplementary figures and images for: Aberrant promoter methylation contributes to LRIG1 silencing in basal/triple-negative breast cancer
Source: Br J Cancer. 2022 Apr 19;127(3):436–48. doi: 10.1038/s41416-022-01812-8 (PMC9346006; doi:10.1038/s41416-022-01812-8)

# SUPPLEMENTAL FIGURE 1

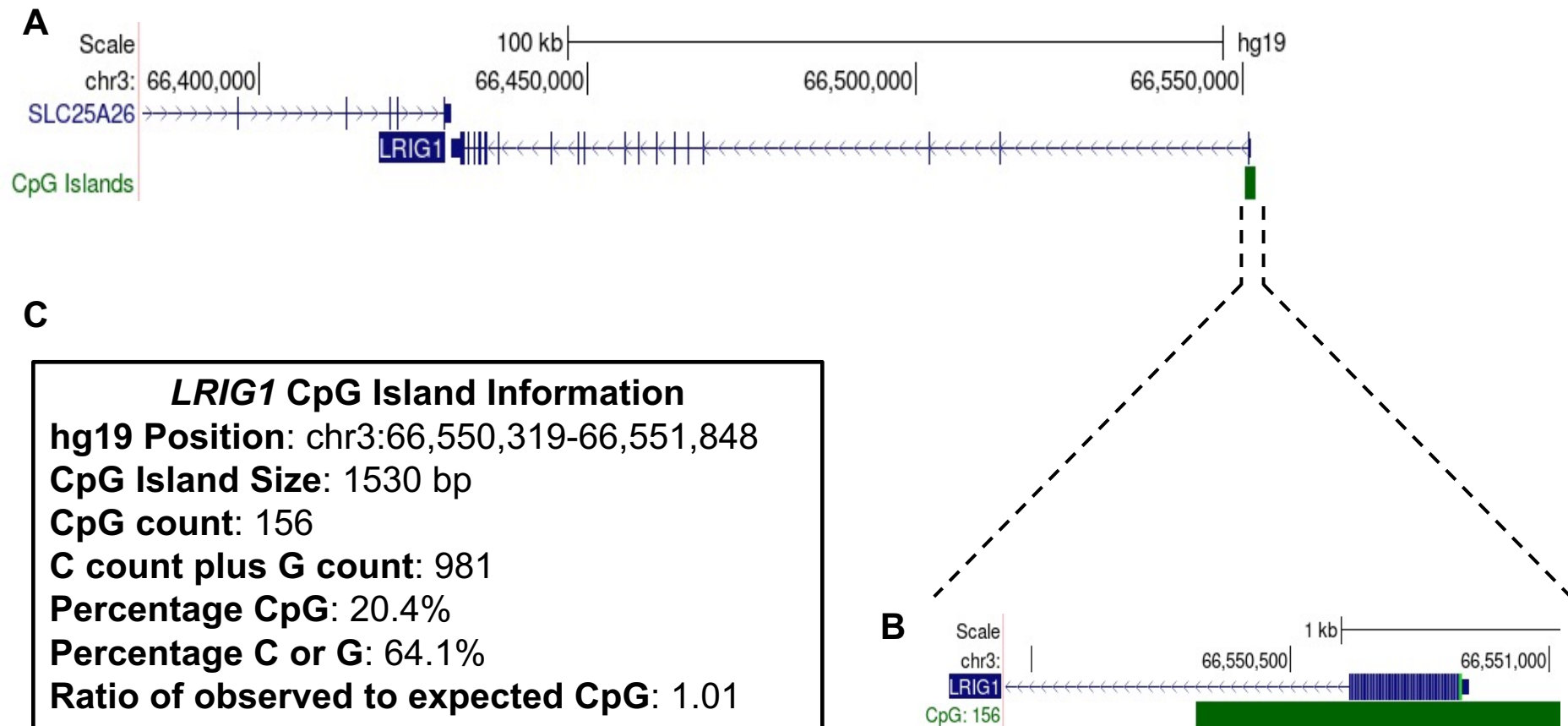

Supplement: Supplementary file 1 — Supplemental Figure 1 [file 41416_2022_1812_MOESM1_ESM.pdf]

# SUPPLEMENTAL FIGURE 2

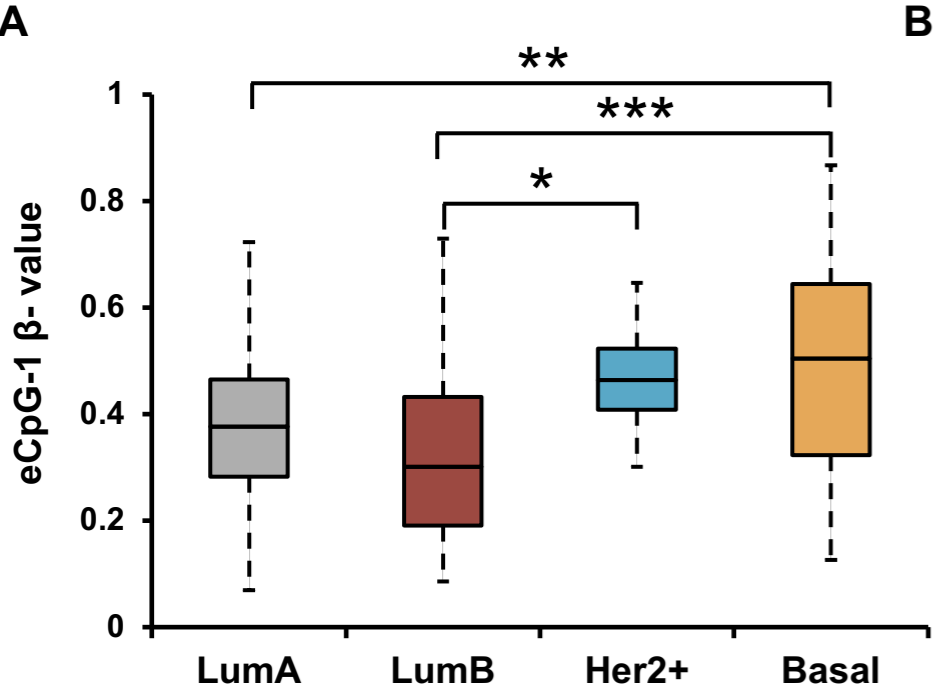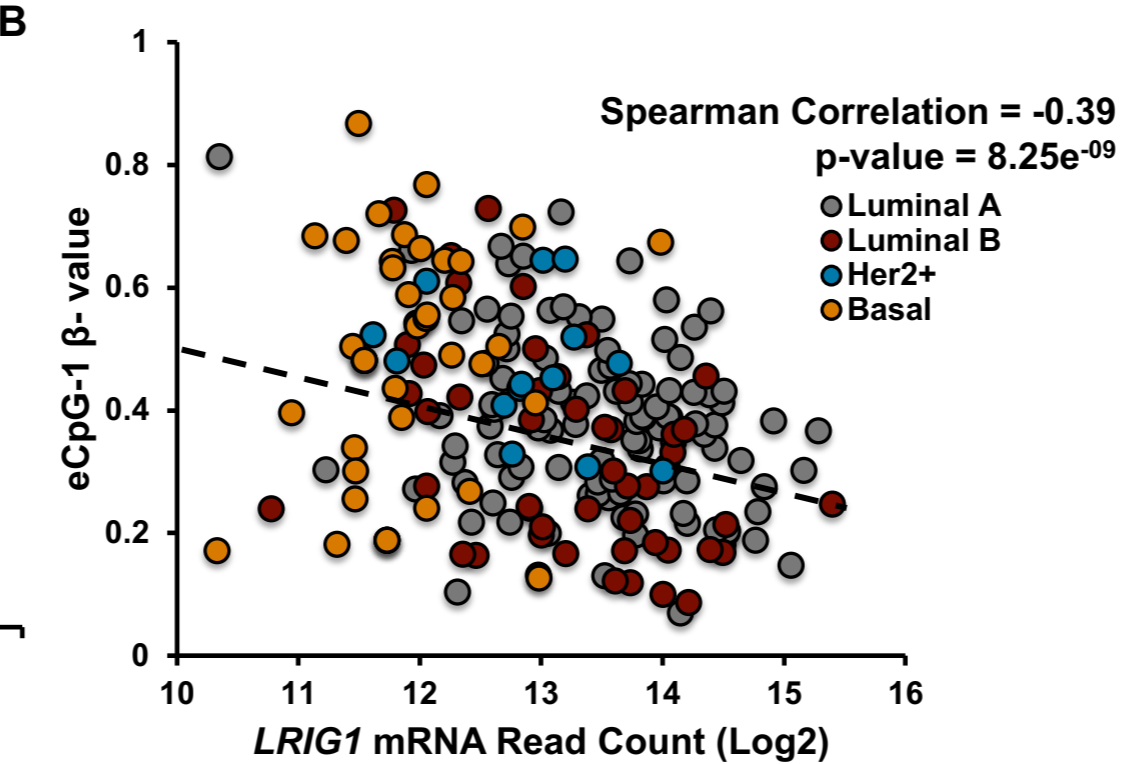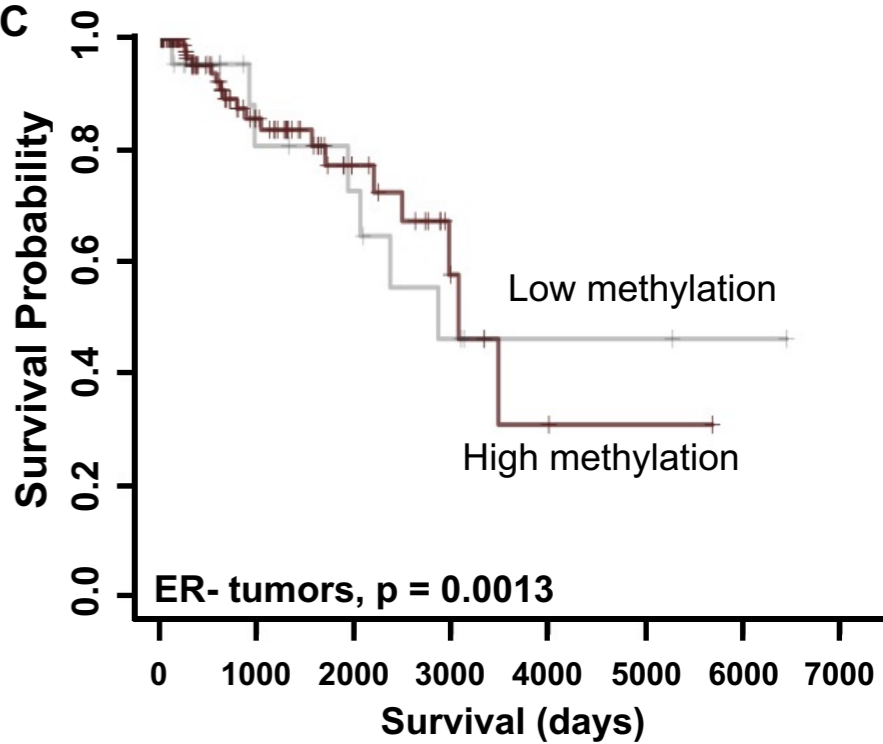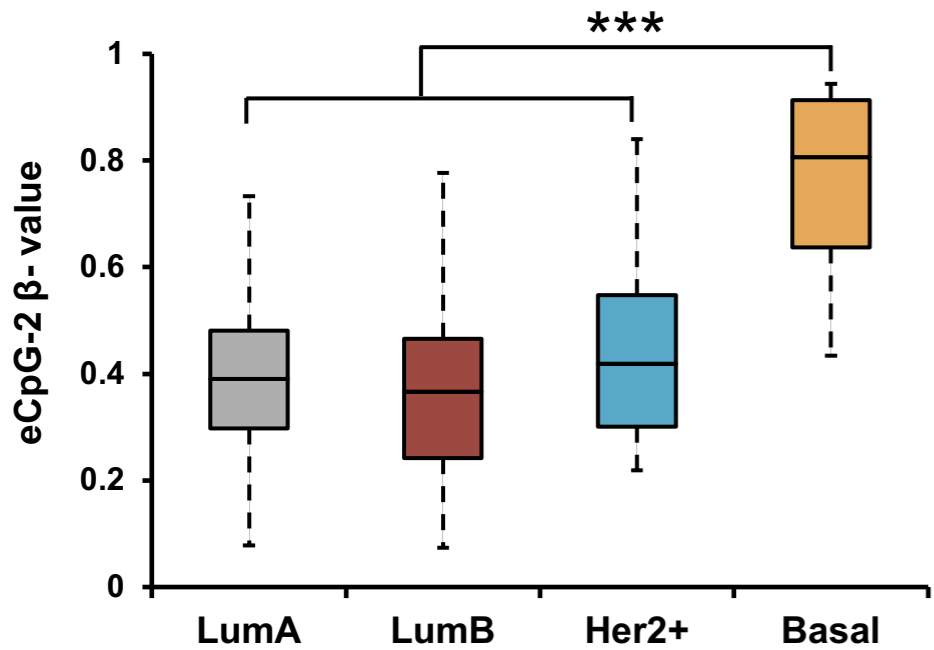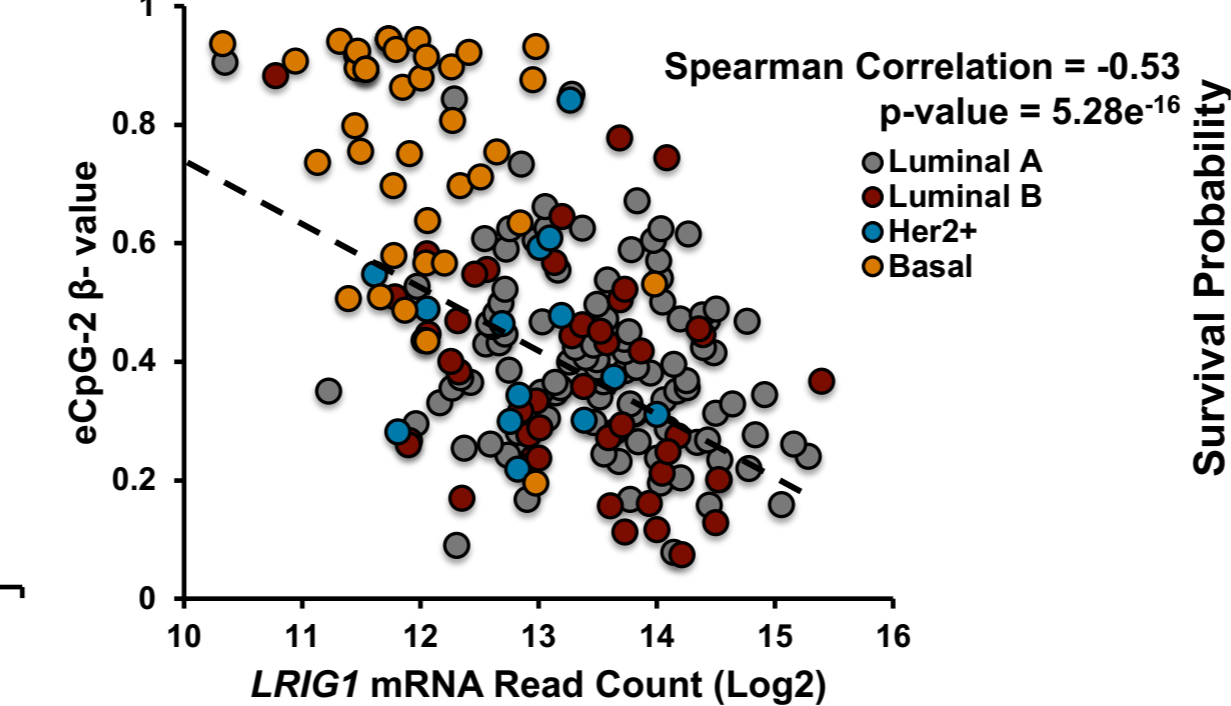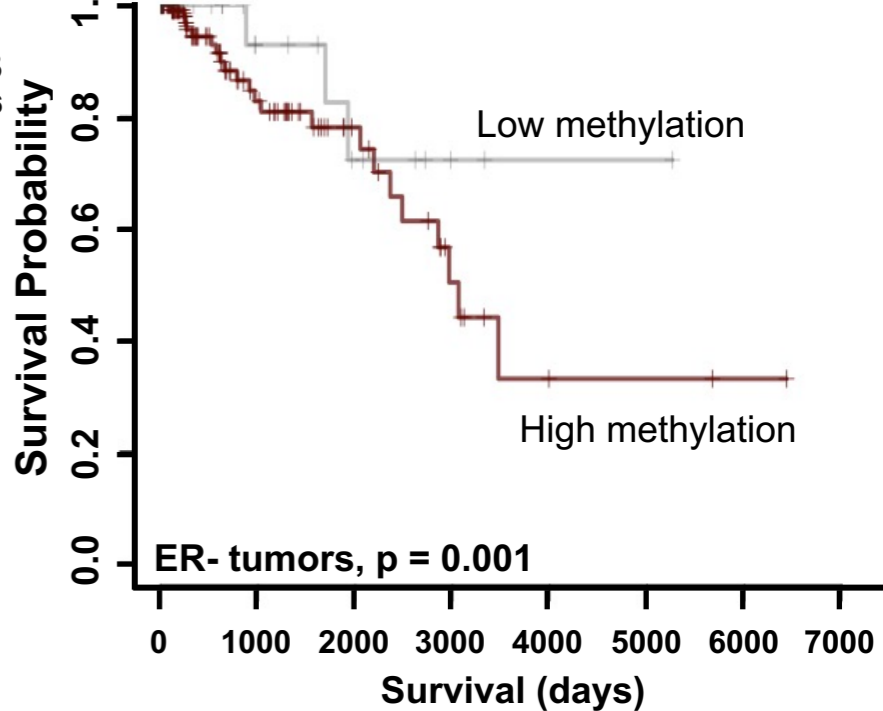

Supplement: Supplementary file 2 — Supplemental Figure 2 [file 41416_2022_1812_MOESM2_ESM.pdf]

# SUPPLEMENTAL FIGURE 3

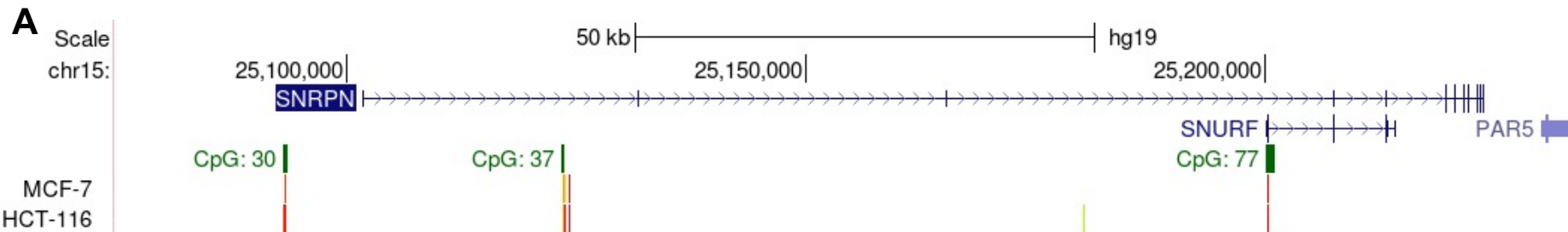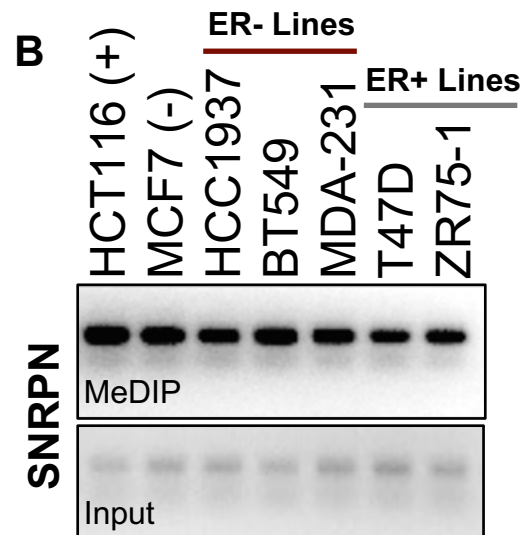

Supplement: Supplementary file 3 — Supplemental Figure 3 [file 41416_2022_1812_MOESM3_ESM.pdf]

# SUPPLEMENTAL FIGURE 4

A

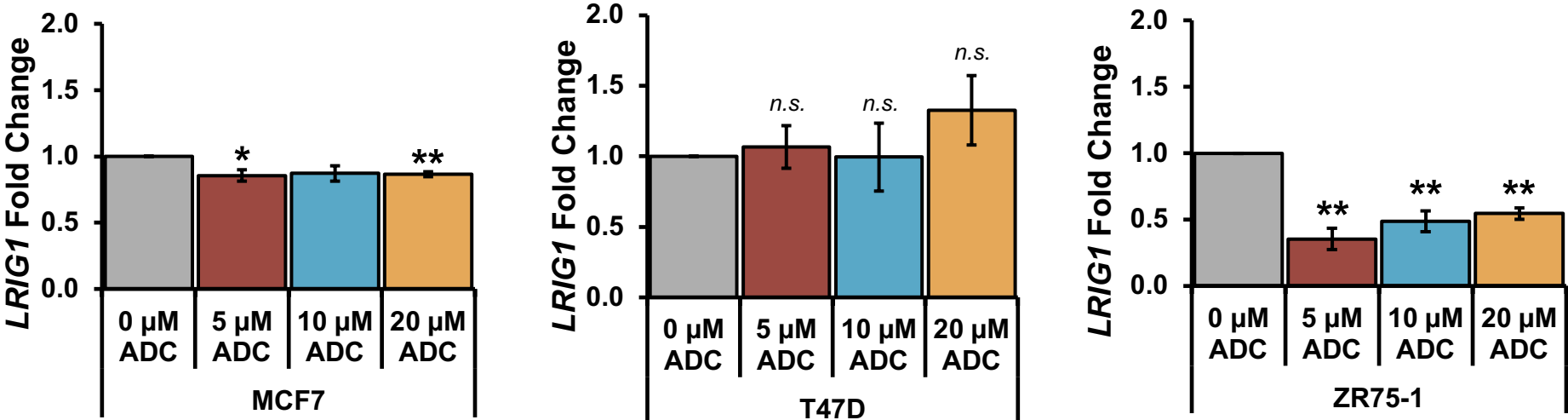

B

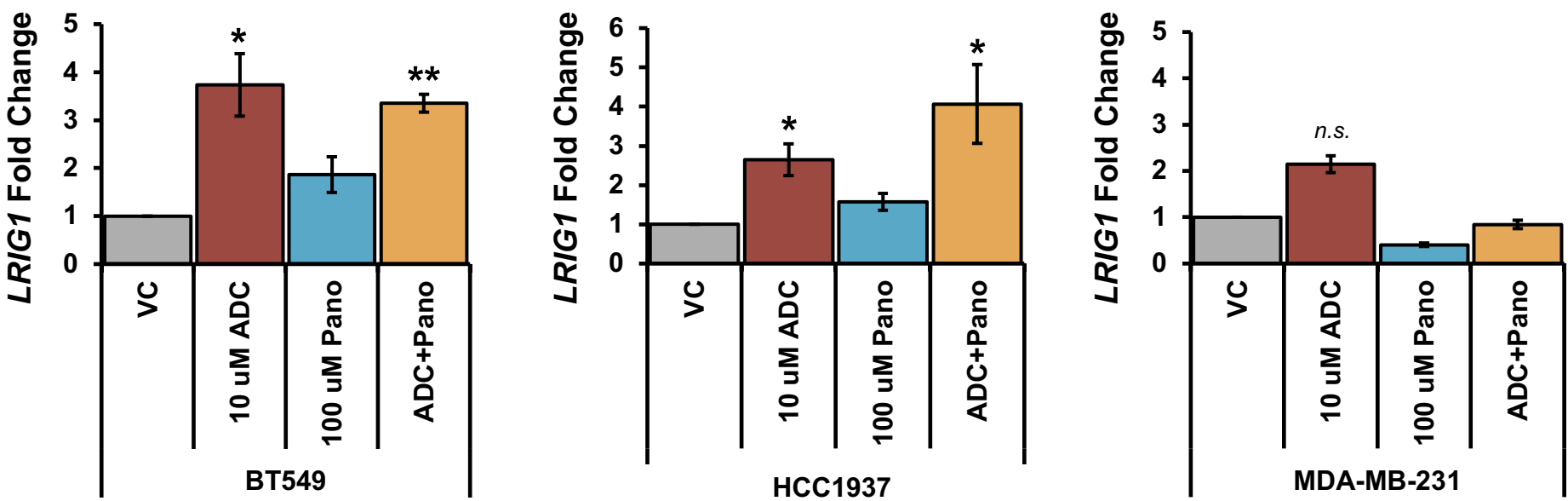

Supplement: Supplementary file 4 — Supplemental Figure 4 [file 41416_2022_1812_MOESM4_ESM.pdf]

# SUPPLEMENTAL FIGURE 5

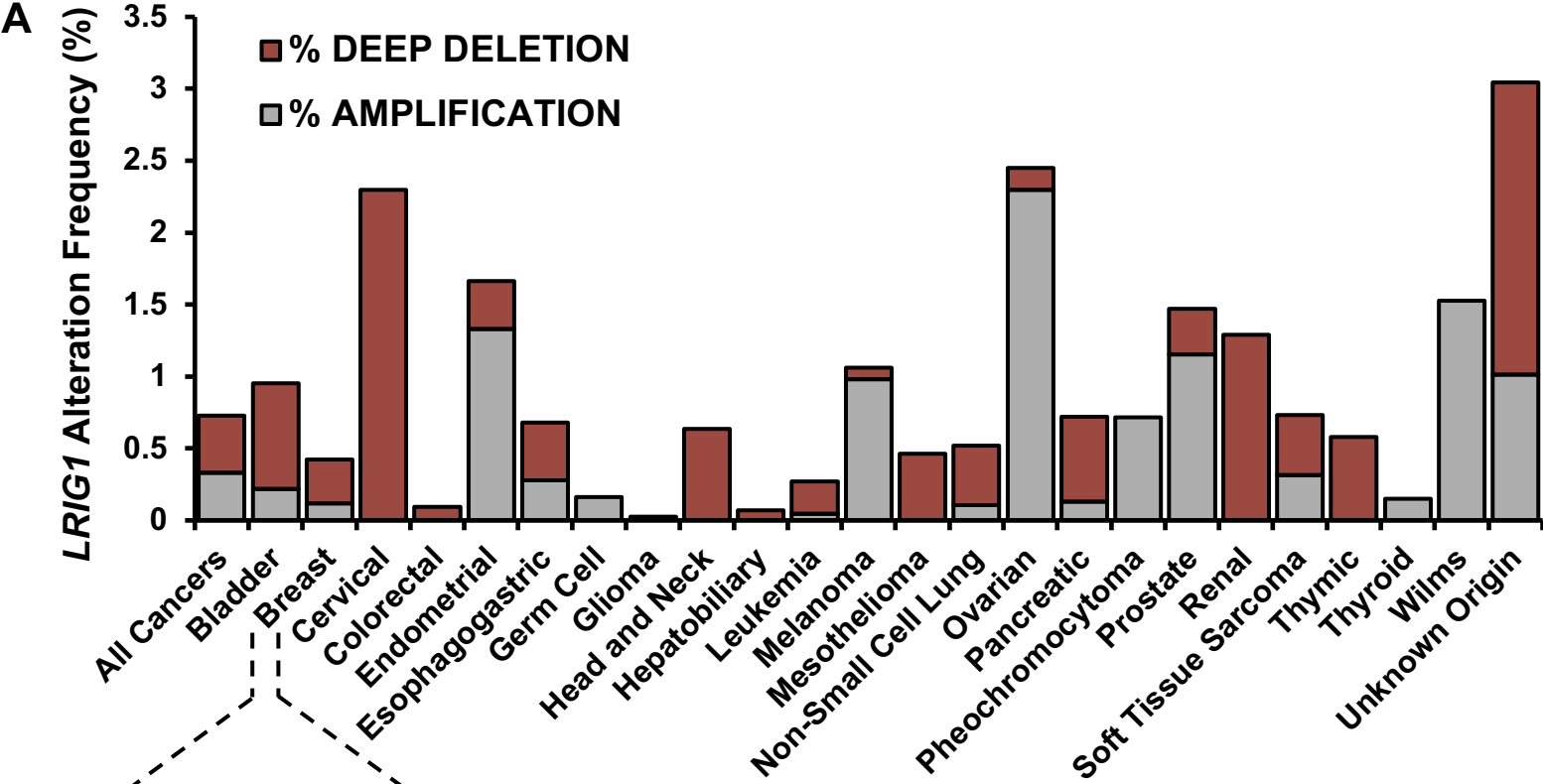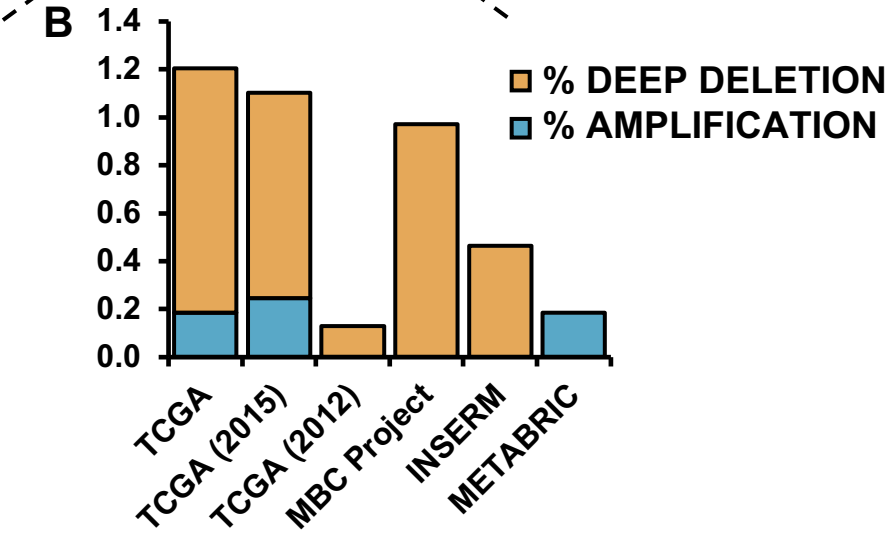

Supplement: Supplementary file 5 — Supplemental Figure 5 [file 41416_2022_1812_MOESM5_ESM.pdf]

SUPPLEMENTAL FIGURE 6

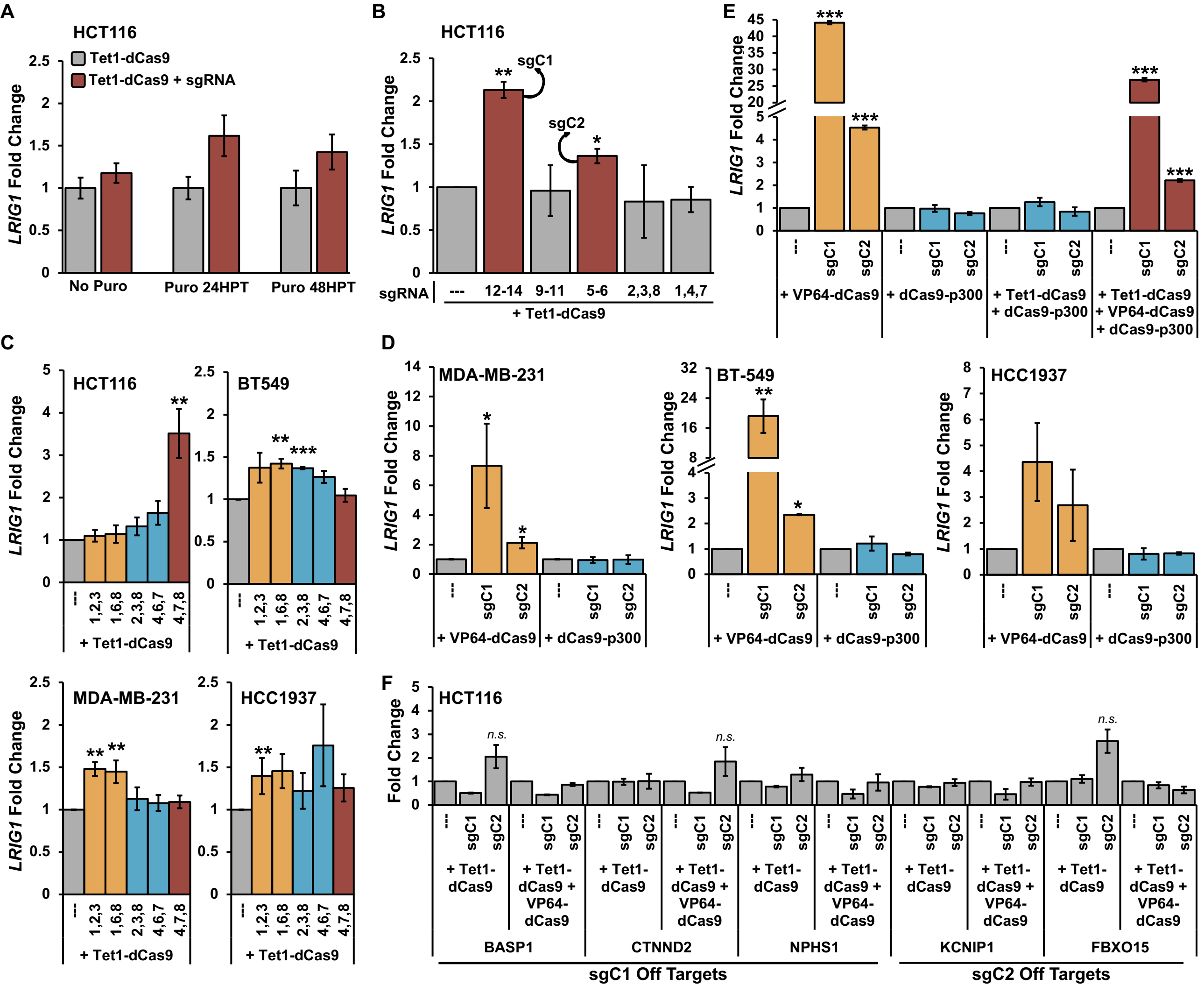

Supplement: Supplementary file 6 — Supplemental Figure 6 [file 41416_2022_1812_MOESM6_ESM.pdf]

SUPPLEMENTAL FIGURE 8

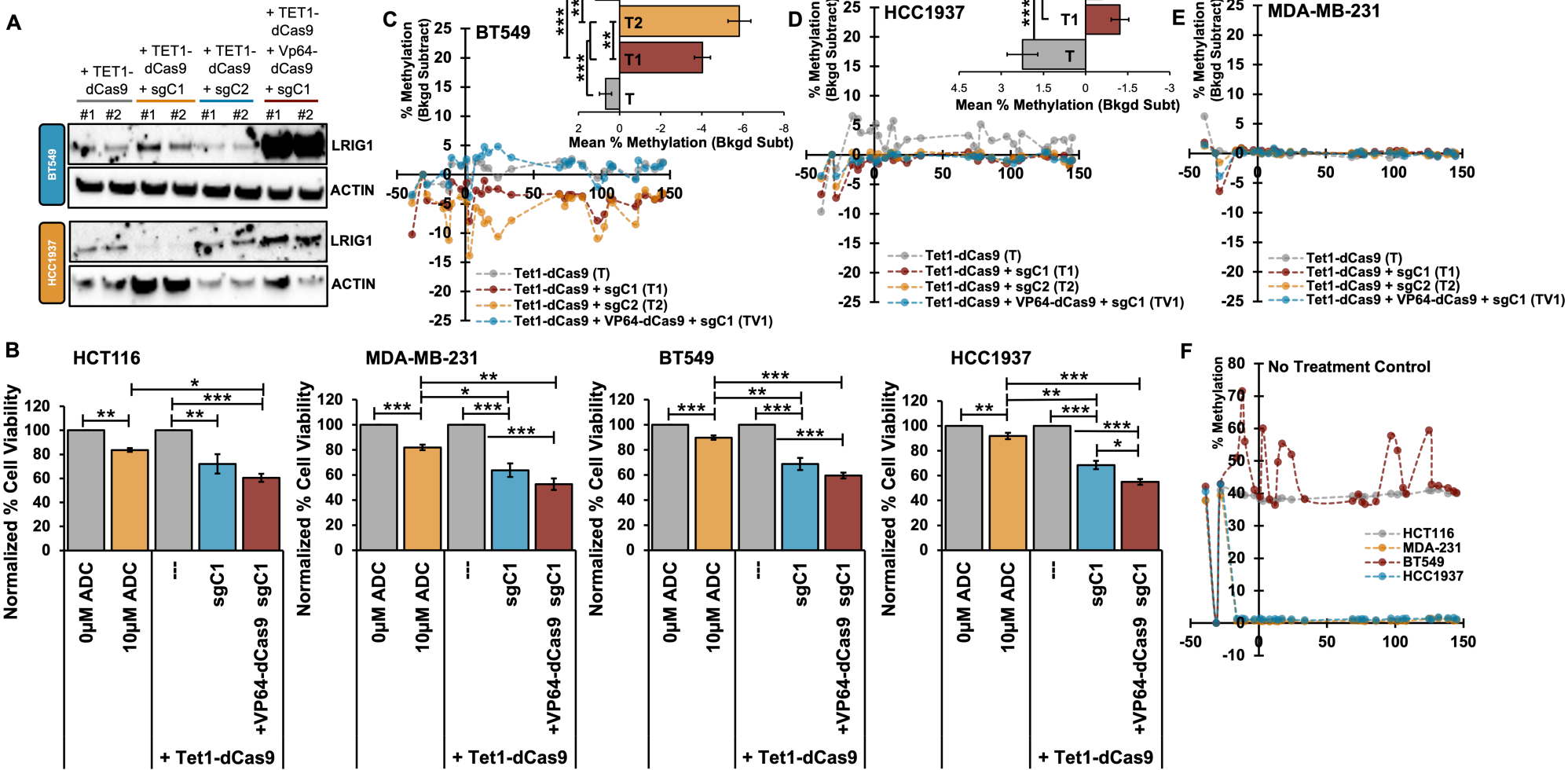

Supplement: Supplementary file 8 — Supplemental Figure 8 [file 41416_2022_1812_MOESM8_ESM.pdf]

# SUPPLEMENTAL FIGURE 9

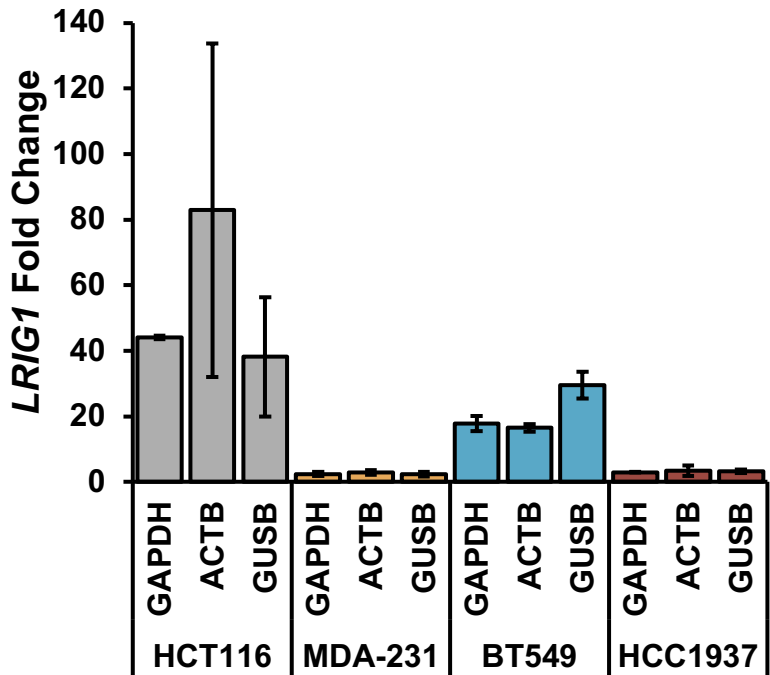

Supplement: Supplementary file 9 — Supplemental Figure 9 [file 41416_2022_1812_MOESM9_ESM.pdf]
