# Supplementary material for: Aberrant promoter methylation contributes to LRIG1 silencing in basal/triple-negative breast cancer
Source: Br J Cancer. 2022 Apr 19;127(3):436–48. doi: 10.1038/s41416-022-01812-8 (PMC9346006; doi:10.1038/s41416-022-01812-8)
Supplement: Supplementary file 10 — Supplemental Table 1 [file 41416_2022_1812_MOESM10_ESM.pdf]

SUPPLEMENTAL TABLE 1

	PAM 50 Subtype	N	*r*	p - value
CpG Island	Luminal A	107	-0.587	$2.95e^{-11}$
	Luminal B	47	-0.588	$1.38e^{-05}$
	Her2+	12	-0.462	0.130
	Basal	34	-0.379	0.027
eCpG-1	Luminal A	107	-0.317	$8.85e^{-04}$
	Luminal B	47	-0.443	0.002
	Her2+	12	-0.362	0.247
	Basal	34	-0.138	0.436
eCpG-2	Luminal A	107	-0.334	0.003
	Luminal B	47	-0.328	0.024
	Her2+	12	-0.059	0.855
	Basal	34	-0.232	0.187
