# Supplementary material for: Aberrant promoter methylation contributes to LRIG1 silencing in basal/triple-negative breast cancer
Source: Br J Cancer. 2022 Apr 19;127(3):436–48. doi: 10.1038/s41416-022-01812-8 (PMC9346006; doi:10.1038/s41416-022-01812-8)
Supplement: Supplementary file 11 — Supplemental Table 2 [file 41416_2022_1812_MOESM11_ESM.pdf]

SUPPLEMENTAL TABLE 2

sgRNA	Target Sequence
sgRNA 1	GCAGCGCGCTCCAGACAAGATGG
sgRNA 2	GGGGACTGTGAGGACCCGAACGG
sgRNA 3	GTGCGGAGTCTAGAGCCGAGCGG
sgRNA 4	CAAGATGGCGCGGCCGGTCCGGG
sgRNA 5	GTCTGGAGACGAGCGAGCGTGGG
sgRNA 6	TCTGTCACTTGGGGATGGAGCGG
sgRNA 7	GCTTTTGCTTCGGCTGGAGCCGG
sgRNA 8	CTCGGCTCTAGACTCCGCACCGG
sgRNA 9	GAACTCCGCCGATTCGGGCAAGG
sgRNA 10	GAGCGCAAAGCCGTAGACCTCGG
sgRNA 11	TTGTTCGGCTGCGAGGTCGGCGG
sgRNA 12	ATCCAACGTGGGCCAGCAGGCGG
sgRNA 13	CCCACCCAAGGTGTGAAGGGCGG
sgRNA14	ACGCCCTCTGCGTCAAGAAGGGG

**PAM sequence is underlined*
