# Supplementary material for: Aberrant promoter methylation contributes to LRIG1 silencing in basal/triple-negative breast cancer
Source: Br J Cancer. 2022 Apr 19;127(3):436–48. doi: 10.1038/s41416-022-01812-8 (PMC9346006; doi:10.1038/s41416-022-01812-8)
Supplement: Supplementary file 12 — Supplemental Figure Legends [file 41416_2022_1812_MOESM12_ESM.docx]

**SUPPLEMENTAL FIGURES LEGENDS**

**Supplemental Figure 1.** **(A)** Snapshot of *LRIG1* gene, and neighboring gene (S*LC25A26*), in the UCSC Genome Browser hg19 build. Vertical tick marks in *LRIG1* gene indicate location of exons 1-19. CpG Island track (green) is present under RefSeq Genes track (blue). **(B)** Zoomed snapshot of UCSC Genome Browser showing CpG island overlap with *LRIG1* transcription start site (TSS, green highlight) and Exon 1. **(C)** *LRIG1* CpG Island information/statistics from UCSC Genome Browser.

**Supplemental Figure 2.** **(A)** Boxplot depicting *LRIG1* β-values at ESR1-enhancer sites, cg24150385 (eCpG-1; top) and cg0971692 (eCpG-2; bottom), in Luminal A (n = 109), Luminal B (n = 47), Her2+ (n = 14), and Basal (n = 41) breast tumors. (Data from TCGA BRCA) **(B)** Scatter plot of patient-matched *LRIG1* mRNA read count and eCpG-1 (top) and eCpG-2 (bottom) β-values in breast tumor samples (n = 209). Black dotted line indicates linear regression. (Data from TCGA BRCA) **(C)** Kaplan-Meier plot of overall survival as a function eCpG-1 (top) and eCpG-2 (bottom) methylation in ER-negative tumors. Patients were segregated into groups based on ERα status and *LRIG1* β-value in their primary tumor. (High methylation/ER-negative: n = 134 (eCpG-1); 133 (eCpG-2), Low methylation/ER-negative: n = 27 (eCpG-1); 28 (eCpG-2)) (Data from TCGA BRCA) *, **, and ***, P < 0.05, P < 0.01, and P < 0.001, respectively; Tukey’s HSD (A), Spearman correlation test (B), log-rank test (C).

**Supplemental Figure 3.** **(A)** Snapshot of *SNRPN* (and *SNURF*) genes in the UCSC Genome Browser hg19 build. RefSeq Genes track (blue). CpG Island track (green). DNA Methylation by Reduced Representation Bisulfite Sequencing (RRBS) track depicts location and levels of methylation in HCT116 and MCF7 cells. Red: 100% methylation, Yellow: 50% methylation, Green: 0% methylation. **(B)** DNA agarose gel of MeDIP-PCR shows methylation levels at the *SNRPN* region across cell lines (n = 7). 5% of input DNA is shown in bottom panel.

**Supplemental Figure 4.** **(A)** Bar plots showing relative *LRIG1* expression levels in ER-positive breast cancer cell lines (MCF7, T47D, ZR75-1) treated with shown concentrations of ADC for 96 hours. Expression was determined from the Ct values using the 2^-ΔΔCt^ method after normalization to *GAPDH*. *LRIG1* fold changes were normalized to 0μM treatments for each cell line, which are set to 1.0. **(B)** Bar plots showing relative *LRIG1* expression levels in BT549, HCC1937, and MDA-MB-231 cells treated with vehicle control (VC), 10μM ADC, 100μM Panobinostat (Pano), or a combination of ADC and Pano at given concentrations. Expression was determined from the Ct values using the 2^-ΔΔCt^ method after normalization to *GAPDH*. *LRIG1* fold changes were normalized to VC values for each cell line, which are set to 1.0. Values are mean ± S.E.M. of three independent experiments. * and **, P < 0.05, P < 0.01, and P < 0.001, respectively, n.s. = not significant (p > 0.05); Student’s t-test (A, B).

**Supplemental Figure 5.** **(A)** Stacked bar plot of *LRIG1* alteration frequency across cancer types (red bars: percentage of tumors with homozygous (deep) deletions of the *LRIG1* gene, gray bars: percentage of tumors with amplifications of the *LRIG1* gene). **(B)** Stacked bar plot of *LRIG1* alteration frequency across six breast cancer studies (yellow bars: percentage of tumors with homozygous (deep) deletions, blue bars: percentage of tumors with amplifications). Data from cBioPortal Copy Number Variation (CNV) analysis.

**Supplemental Figure 6.** **(A)** Bar plot showing relative *LRIG1* expression levels in HCT116 cells transfected with Tet1-dCas9 alone or Tet1-dCas9 with guide RNAs, followed by no selection, or selection with Puromycin at 24- or 48-hours post transfection (HPT). **(B)** Bar plot showing relative *LRIG1* expression levels in HCT116 cells transfected with Tet1-dCas9 or Tet1-dCas9 with indicated sgRNA combinations. **(C)** Bar plots showing relative LRI*G1* expression levels in HCT116, MDA-MB-231, BT549, and HCC1937 cells transfected with Tet1-dCas9 alone or Tet1-dCas9 with indicated sgRNA combinations. **(D)** Bar plots showing relative *LRIG1* expression levels in MDA-MB-231, BT549, and HCC1937 cells transfected with VP64-dCas9 or dCas9-p300 (Core) alone or with guide RNA combinations (sgC1, sgC2). **(E)** Bar plot showing relative *LRIG1* expression levels in HCT116 cells transfected with indicated dCas9s complexes with or without guide RNA combinations (sgC1, sgC2). **(F)** Bar plot showing relative expression levels of putative guide RNA off-targets (sgC1: *BASP1*, *CTNND*2, *NPHS1*; sgC2: *KCNIP1*, *FBXO15*) in HCT116 cells transfected with Tet1-dCas9 alone or with guide RNA combinations (sgC1, sgC2), and Tet1-dCas9/VP64-dCas9 alone or with guide RNA combinations (sgC1, sgC2). For A – F, expression was determined from the Ct values using the 2^-ΔΔCt^ method after normalization to *GAPDH*. *LRIG1* fold changes were normalized to no sgRNA values for each group, which are set to 1.0. Values are mean ± S.E.M. of two (B, D, F) or three (C, E) independent experiments. *, **, and ***, P < 0.05, P < 0.01, and P < 0.001, respectively, n.s. = not significant (p > 0.05); Student’s t-test (A – F).

**Supplemental Figure 7.** Visualization of the predicted off-target binding sites for *LRIG1*. For each single guide RNA, the letters at the top show the nucleotide sequence for the *LRIG1* on-target binding site (not including the NGG corresponding to the PAM site). Below are off-target sequences predicted to occur for at least two sgRNAs (Combination 1), and for both sgRNAs (Combination 2). Colored letters correspond to single nucleotide mismatches. Black dots indicate matches to the sgRNA/on-target sequence.

**Supplemental Figure 8. (A)** LRIG1 protein abundance in BT549 and HCC1937 cells transfected with Tet1-dCas9 alone, Tet1-dCas9 with sgC1 or sgC2, and a combination of Tet1-dCas9 and VP64-dCas9 with sgC1, as assessed by Western Blotting. Actin serves as a loading control. **(B)** Bar plot of normalized percent viability in HCT116, MDA-MB-231, BT549, and HCC1937 cells treated with 0μM or 10μM ADC, transfected with Tet1-dCas9 alone, Tet1-dCas9 with sgC1, or a combination of Tet1-dCas9 and VP64-dCas9 with sgC1. Percent cell viability was determined after normalizing to 0μM ADC or no sgRNA values for each group. **(C-F)** Line plots of change in percent methylation in BT549 **(C)**, HCC1937 **(D)**, and MDA-MB-231 **(E)** cells transfected with Tet1-dCas9, Tet1-dCas9 with guide RNA combinations (sgC1, sgC2), or a combination of Tet1-dCas9 and VP64-dCas9 with sgC1, as assessed by targeted sequencing of bisulfite converted genomic DNA across 30 CpG dinucleotides near the *LRIG1* transcriptional start site. Change in percent methylation was determined after background subtracting the no treatment control percent methylation values **(F)**. X-axis depicts CpG distances from *LRIG1* transcriptional start site (at 0). Values are mean ± S.E.M. of two (B-C, E-F), three (A), or four (D) independent experiments. *, **, and ***, P < 0.05, P < 0.01, and P < 0.001, respectively; Student’s t-test (B).

**Supplemental Figure 9.** Bar plot showing relative *LRIG1* expression levels in treated or transfected cells using *GAPDH*, *ACTB*, or *GUSB* as qPCR reference genes. Expression was determined from the Ct values using the 2^-ΔΔCt^ method after normalization to respective reference genes. *LRIG1* fold changes were normalized to no control values for each group, which were set to 1.0 (not shown). Values are mean ± S.E.M. of two independent experiments.
