# Supplementary material for: Aberrant promoter methylation contributes to LRIG1 silencing in basal/triple-negative breast cancer
Source: Br J Cancer. 2022 Apr 19;127(3):436–48. doi: 10.1038/s41416-022-01812-8 (PMC9346006; doi:10.1038/s41416-022-01812-8)

SUPPLEMENTAL FIGURE 7

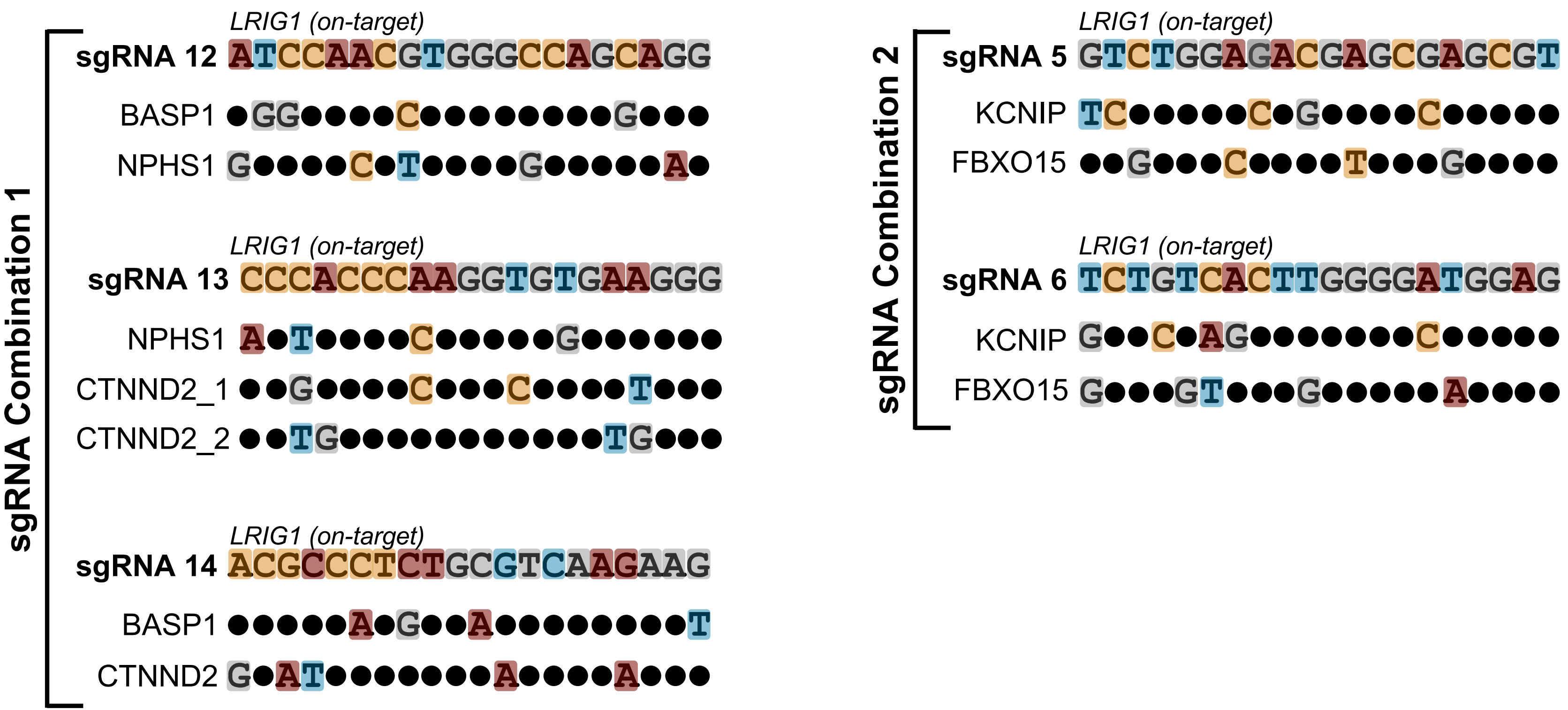

Supplement: Supplementary file 7 — Supplemental Figure 7 [file 41416_2022_1812_MOESM7_ESM.pdf]
